# CD74 is a functional MIF receptor on activated CD4^+^ T cells

**DOI:** 10.1007/s00018-024-05338-5

**Published:** 2024-07-11

**Authors:** Lin Zhang, Iris Woltering, Mathias Holzner, Markus Brandhofer, Carl-Christian Schaefer, Genta Bushati, Simon Ebert, Bishan Yang, Maximilian Muenchhoff, Johannes C. Hellmuth, Clemens Scherer, Christian Wichmann, David Effinger, Max Hübner, Omar El Bounkari, Patrick Scheiermann, Jürgen Bernhagen, Adrian Hoffmann

**Affiliations:** 1 Division of Vascular Biology, Institute for Stroke and Dementia Research (ISD), LMU University Hospital (LMU Klinikum), Ludwig-Maximilians-Universität (LMU) München, Feodor-Lynen-Straße 17, 81377 Munich, Germany; 2 Max von Pettenkofer Institute and Gene Center, Virology, National Reference Center for Retroviruses, Ludwig-Maximilians-Universität (LMU) Munich, Munich, Germany; 3 German Center for Infection Research (DZIF), Partner Site Munich, Munich, Germany; 4 COVID-19 Registry of the LMU Munich (CORKUM), LMU University Hospital, Ludwig-Maximilians-Universität (LMU) Munich, Munich, Germany; 5 Department of Medicine III, LMU University Hospital, Ludwig-Maximilians-Universität (LMU) Munich, Munich, Germany; 6 Department of Medicine I, LMU University Hospital, Ludwig-Maximilians-Universität (LMU) Munich, Munich, Germany; 7 Division of Transfusion Medicine, Cell Therapeutics and Haemostaseology, LMU University Hospital, Ludwig-Maximilians-Universität (LMU) Munich, Munich, Germany; 8 Department of Anaesthesiology, LMU University Hospital, Ludwig-Maximilians-Universität (LMU) Munich, Marchioninistraße 15, 81377 Munich, Germany; 9 Walter Brendel Centre of Experimental Medicine, Ludwig-Maximilians-Universität (LMU) Munich, Munich, Germany; 10 German Centre of Cardiovascular Research (DZHK), Partner Site Munich Heart Alliance, Munich, Germany

**Keywords:** CD74/invariant chain, Macrophage migration inhibitory factor, MIF, T cells, Atypical chemokine, CXCR4

## Abstract

Next to its classical role in MHC II-mediated antigen presentation, CD74 was identified as a high-affinity receptor for macrophage migration inhibitory factor (MIF), a pleiotropic cytokine and major determinant of various acute and chronic inflammatory conditions, cardiovascular diseases and cancer. Recent evidence suggests that CD74 is expressed in T cells, but the functional relevance of this observation is poorly understood. Here, we characterized the regulation of CD74 expression and that of the MIF chemokine receptors during activation of human CD4^+^ T cells and studied links to MIF-induced T-cell migration, function, and COVID-19 disease stage. MIF receptor profiling of resting primary human CD4^+^ T cells via flow cytometry revealed high surface expression of CXCR4, while CD74, CXCR2 and ACKR3/CXCR7 were not measurably expressed. However, CD4^+^ T cells constitutively expressed CD74 intracellularly, which upon T-cell activation was significantly upregulated, post-translationally modified by chondroitin sulfate and could be detected on the cell surface, as determined by flow cytometry, Western blot, immunohistochemistry, and re-analysis of available RNA-sequencing and proteomic data sets. Applying 3D-matrix-based live cell-imaging and receptor pathway-specific inhibitors, we determined a causal involvement of CD74 and CXCR4 in MIF-induced CD4^+^ T-cell migration. Mechanistically, proximity ligation assay visualized CD74/CXCR4 heterocomplexes on activated CD4^+^ T cells, which were significantly diminished after MIF treatment, pointing towards a MIF-mediated internalization process. Lastly, in a cohort of 30 COVID-19 patients, CD74 surface expression was found to be significantly upregulated on CD4^+^ and CD8^+^ T cells in patients with severe compared to patients with only mild disease course. Together, our study characterizes the MIF receptor network in the course of T-cell activation and reveals CD74 as a novel functional MIF receptor and MHC II-independent activation marker of primary human CD4^+^ T cells.

**Supplementary Information:**

The online version contains supplementary material available at 10.1007/s00018-024-05338-5.

## Introduction

CD74, also known as major histocompatibility complex class II (MHC II) invariant chain (Ii), is a type II transmembrane glycoprotein that plays a crucial role in MHC II-mediated antigen presentation mainly by acting as a class II chaperone [1]. Accordingly, CD74 expression is seen in antigen-presenting B cells, monocytes/macrophages, and dendritic cells. Beyond this canonical function, CD74 was discovered as a high affinity receptor for the cytokine and atypical chemokine MIF that has emerged as an upstream regulatory and inflammatory mediator in the pathogenesis of various cardiovascular, infectious, autoimmune and cancerous diseases [2–5]. Next to CD74, the currently known MIF receptors comprise the classical chemokine receptors CXCR2, CXCR4 and ACKR3/CXCR7. These are found to a varying degree on nearly all leukocyte subsets enabling MIF to shape the local immune cell profile in inflamed tissues [3, 4, 6–8]. In-depth investigations of the underlying molecular mechanisms including the detailed characterization of ligand/receptor interactions not only placed MIF in this complex ligand/receptor network, but also enabled the development of various MIF-targeted treatment strategies [9, 10].

MIF-mediated signaling via CD74 has been shown to be dependent on receptor complex formation with CD44, CXCR2, CXCR4 and ACKR3/CXCR7, inducing downstream phosphatidylinositol 3-kinase/protein kinase B (PI3K/Akt), adenosine monophosphate-activated protein kinase (AMPK), nuclear factor-κB (NF-κB), calcium signaling, and extracellular signal-regulated kinase (ERK) pathways [4, 8, 11, 12]. Thereby, CD74 is critically involved in MIF-driven immune cell recruitment and activation of a variety of cellular responses, including cell proliferation and cell metabolism that have been found to play a role in cancer, metabolic and ischemic heart disease [4, 5, 13–17].

In T cells, MIF was previously shown to be secreted upon activation and to influence key immunological processes such as migration, proliferation, apoptosis and to promote a Th17-phenotype [18–24]. MIF-receptor pathways have been amply studied in numerous cell types, but despite its first description as a soluble T cell-derived mediator more than 50 years ago, our current understanding of the receptor mechanisms triggered by MIF in human T cells is still incomplete [25]. In particular, with only very few incidental descriptive reports on CD74 expression in human T cells available, the role of CD74 receptor activity in T cells is unclear. In fact, although CD74 upregulation in the context of inflammation and cell stress has previously been observed in MHC II-negative cell types such as endothelial cells, cancer cells, or cardiomyocytes, the occurrence of CD74 in T cells is surprising, as T cells, which are MHC class II-negative themselves, are best known for their role in MHC-based peptide recognition from MHC-II^+^ antigen-presenting immune cells [21, 26–28]. Therefore, this study aimed to characterize the regulation of CD74 and its relevance for MIF-mediated functions in human CD4^+^ T cells in the course of T-cell activation, with CD4^+^ T cells representing the cornerstone of the adaptive immune system by mediating immune homeostasis, antigen-recognition, self-tolerance and immunological memory. CD4^+^ T-cell activation occurs through binding of the T-cell receptor (TCR) to an MHC II-bound antigen in the presence of costimulatory signals and represents the crucial mechanism by which T cells respond to foreign or endogenous antigens and differentiate into effector T cells [29].

Here, we provide evidence that CD4^+^ T cells constitutively express CD74 intracellularly, which upon T-cell activation, is significantly and rapidly upregulated, post-translationally modified by chondroitin sulfate (CS) and translocated to the cell surface to fulfil its function as MIF receptor. By exploiting flow cytometry, Western blot (WB), immunohistochemistry, and re-analysis of published RNA-sequencing (RNAseq) and proteomic data sets, our study identified CD74 as a novel activation marker of T cells that is regulated independent of MHC II. Functional studies revealed a significant involvement of both CD74 and CXCR4 in MIF-elicited CD4^+^ T-cell chemotaxis. Proximity ligation assay (PLA) visualized CD74/CXCR4 complexes on activated T cells, which are internalized upon MIF-treatment.

With accumulating evidence pointing towards a critical role of MIF as a prognostic marker to predict disease severity and patient outcome in COVID-19 and observations of an impaired T cell response during Sars-CoV-2 infections often displayed by sustained T-cell activation, we aimed to confirm the translational relevance of our findings in the context of COVID-19 [30–32]. In a patient cohort of 30 patients with mild and severe COVID-19, we observed a significant upregulation of CD74 surface expression on CD4^+^ and CD8^+^ T cells in patients with severe (WHO grade ≥ 5) compared to patients with only mild disease (WHO grade 1–3), which was accompanied by CD74 upregulation on classical monocytes. Together, our data characterize CD74 as a relevant MHC II-independent functional MIF-receptor in activated human T cells.

## Materials and methods

### Proteins and reagents

Biologically active and endotoxin-free recombinant human MIF was prepared as previously described [9, 33]. Briefly, recombinant MIF was obtained by expression in the pET11b/*E. coli* BL21/DE3 system, followed by recovery of the supernatant of the bacterial lysate, centrifugation, filtration, purification by Mono Q anion exchange and C8 reverse-phase chromatography, as well as dialysis-based renaturation. The protein as purified by this procedure is essentially endotoxin-free (< 10–15 pg/µg) and exhibits a purity grade of ~ 98% as determined by SDS/PAGE/silver staining [9, 33].

### Isolation of human peripheral blood-derived leukocyte subsets

Peripheral blood mononuclear cells (PBMCs) were isolated by density gradient centrifugation using Ficoll-Paque Plus (GE Healthcare, Freiburg, Germany) from peripheral blood (1:3 mixture with PBS) that was collected in conical chambers of a Leukoreduction System (LRS) during thrombocyte apheresis of anonymous and healthy thrombocyte donors at the Division of Transfusion Medicine, Cell Therapeutics and Haemostaseology of the LMU University Hospital. Red blood cells (RBCs) were lysed using RBC lysis buffer (BioLegend, San Diego, USA) for 3 min at room temperature (RT). Subsequently, cells were washed with RPMI 1640 media (Gibco, Karlsruhe, Germany) and supplemented with 10% fetal bovine serum (FBS). Human CD4^+^ T cells were isolated by negative depletion from the enriched PBMC fraction using the human CD4^+^ T-cell isolation kit from Miltenyi Biotec (Bergisch Gladbach, Germany) according to the manufacturer’s instructions. The purity of isolated CD4^+^ T cells was analyzed by flow cytometry using anti-CD3 and anti-CD4 antibodies and estimated to be 95–98% (Supp. Fig. 1A).

Human neutrophilic granulocytes were isolated from blood that was obtained from healthy human volunteers with informed consent by dextran sedimentation followed by a density gradient centrifugation using Ficoll-Paque Plus. Cells were cultivated in RPMI 1640 medium supplemented with 10% FBS, 1% penicillin/streptomycin in a cell culture incubator at 37 °C and 5% CO_2_. Studies abide by the Declaration of Helsinki principles and were approved by ethics approvals 18-104 and 23-0639 of the Ethics Committee of LMU Munich, which encompasses the use of anonymized tissue and blood specimens for research purposes.

### Analysis of human COVID-19 clinical specimens

PBMCs that were purified by density centrifugation (Histopaque 1077 from Sigma-Aldrich, St. Louis, USA) from 30 patients with PCR-verified COVID-19 infection were obtained from the COVID-19 Registry of the LMU University Hospital Munich (CORKUM, WHO trial ID DRKS00021225). The study was approved by the local ethical committee of the University Hospital (project numbers: 20-245 and 23-0711) and was conducted according to the Guidelines of the World Medical Association Declaration of Helsinki. All patients provided informed consent. Baseline information like age, gender and laboratory status was provided. Patients were classified according to ordinal scale for clinical improvement of COVID-19 infection reported by the WHO (Blueprint W. Novel Coronavirus. COVID-19 Therapeutic Trial Synopsis. 2020. https://www.who.int/blueprint/priority-diseases/key-action/COVID-19_Treatment_Trial_Design_Master_Protocol_synopsis_Final_18022020pdf (accessed on 5 February 2021) [Internet] Available from: https://bsitd.com.bd/wp-content/uploads/2020/06/7_an-international-randomised-trial-of-candidate-vaccines-against-covid-19.pdf.) and grouped into two sub-cohorts based on disease severity in mild (18 patients, WHO grade I–III, mean age of 59.39 years ± 18.24 years, 5 female and 13 male patients) and severe disease (12 patients, WHO grade ≥ V, mean age of 67.50 years ± 11.26 years, 4 female and 8 male patients). Due to heterogeneity of available time-points for each patient, we chose the time-point closest to admission to the hospital. Using inflammation markers C-reactive protein (CRP) and Interleukin 6 (IL-6), we identified the inflammation peak for each patient, defined as the highest measured CRP or IL-6 value. Human CD3^+^ T cells were isolated by positive depletion from the enriched PBMC fraction using CD3^+^ microbeads from Miltenyi Biotec (Bergisch Gladbach, Germany) according to the manufacturer’s instructions. CXCR4 and CD74 expression was determined in CD3^+^-selected cells that were further characterized by CD4, CD8, and HLA-DR surface expression and CD3^−^-selected cells after identification of monocyte subpopulations by CD14, CD16 and HLA-DR surface expression as described by Marimuthu et al. via flow cytometry using a FACS Canto II (BD Biosciences, Franklin Lakes, USA). Quantification was performed using FlowJo V10 software, version 10.2 (Tree Star, Ashland, USA). (Supp. Figure 1B and 1C, Supp. Table 1) [34].

### In vitro activation of peripheral blood-derived CD4^+^ T cells

When indicated, purified CD4^+^ T cells were cultivated and in vitro-activated using anti-CD3/CD28-coated magnetic beads (Dynabeads™ Human T Activator, ThermoFisher, Waltham, USA) for different time periods according to the manufacturer’s protocol with a bead to cell ratio of 1:1.5 for flow cytometry experiments and 1:4 for WB, immunohistochemistry and functional studies. For following experiments, the activation beads were removed using magnetic separation.

### Flow cytometry

The cell surface expression of immune cell markers or MIF receptors was analyzed by flow cytometry using antibodies directed against CD3, CD4, CD8, CD45RO/RA, CD74, CXCR4 or HLA-DR (details in Supp. Table 1). In brief, 2 × 10^5^ cells were washed three times with ice-cold PBS supplemented with 0.5% BSA and then incubated with the above-mentioned antibodies for 1 h at 4 °C in the dark. For intracellular staining, cells were fixed and permeabilized using intracellular fixation and permeabilization buffer (ThermoFisher). After incubation, cells were washed thoroughly and analyzed using a BD FACSVerse™ (BD Biosciences). Quantification was performed using FlowJo V10 software, version 10.2 (Tree Star).

### SDS-PAGE and Western blot

For WB analysis, cells were washed three times with PBS and resuspended in Pierce™ RIPA lysis and extraction buffer (ThermoFisher). Protein concentrations of the according cell lysates were determined using the Pierce™ BCA protein assay kit (ThermoFisher) and an EnSpire plate reader (PerkinElmer, Waltham, USA) according to the manufacturer’s protocol. Samples were diluted in LDS sample buffer (NuPAGE, ThermoFisher), boiled at 95°C for 15 min and equal amounts of protein were loaded onto 10% SDS–polyacrylamide gels (NuPAGE, ThermoFisher) and transferred to polyvinylidene difluoride (PVDF) membranes (Carl Roth, Karlsruhe, Germany). The CozyHi prestained protein ladder (highqu, Kraichtal, Germany) was used as a protein size marker. For antigen detection, membranes were blocked in PBS-Tween-20 containing 5% BSA (Roth) for 1 h and subsequently incubated overnight at 4 °C with the primary antibodies anti-β-actin (sc-47778, 1:1000, Santa Cruz, Dallas, Texas, USA) or anti-CD74 (LN1, 555317, 1:500, BD Biosciences) diluted in blocking buffer. On the next day, membranes were washed and incubated with the HRP-linked secondary antibody goat anti-mouse IgG2a (ab97245, abcam, Cambridge, UK) or goat anti-rat IgG (HAF005, R&D Systems, Minneapolis, USA). To reveal protein content, signals were detected by chemiluminescence on an Odyssey^®^ Fc Imager (LI-COR Biosciences GmbH, Bad Homburg, Germany) using SuperSignal™ West Dura ECL substrate (ThermoFisher).

### Chondroitinase treatment

To specifically cleave CS modifications of protein in 72 h-activated CD4^+^ T cells, cells were washed with PBS and resuspended in chondroitinase buffer (50 mM Tris–HCl, pH 8.0, 50 mM sodium acetate). Cells were lysed by 5 min of sonication in a water bath (Elmasonic S 40, Elma Schmidbauer GmbH, Singen, Germany), followed by brief homogenization using steel beads in a bead mill at 50 Hz (TissueLyser LT, QIAGEN, Hilden, Germany). To cleave CS from proteins, chondroitinase ABC from *Proteus vulgaris* (Sigma-Aldrich / Merck KgaA, Darmstadt, Germany) was added to a concentration of 0.6 U/ml. Samples were incubated for 2 h at the enzyme’s temperature optimum of 37°C and directly prepared for analysis via SDS-PAGE and WB.

### Re-analysis of RNA-seq and mass spectrometry datasets

For analysis of mRNA expression levels, single cell RNA-seq data published by Szabo et al. were re-analyzed [35]. The data is publicly available on the gene expression omnibus (GEO) with Accession Number GSE126030. Plots were generated using the Single Cell Expression Atlas of the European Bioinformatics Institute (EBI) of the European Molecular Biology Laboratory (EMBL) (https://www.ebi.ac.uk/gxa/sc/experiments/E-HCAD-8/results/tsne, last visited 20th of December, 2023). Secondly, a bulk-RNAseq data set together with the according proteomic data as recently published by Cano-Gamez et al. was re-analyzed [28]. The RNAseq raw data were accessed via the Open Targets website (https://www.opentargets.org/projects/effectorness). Differential gene expression (DEG) analysis between the conditions was performed using R version 4.3.2 and the DESeq2 package [36]. Subsequently, differentially expressed genes (DEGs) were visualized using an EnhancedVolcano plot and ggplot2 [37, 38]. The full analysis code is published on GitHub (https://github.com/SimonE1220/CD74Tcelldiff). The available proteomic raw data were accessed via the Proteomics Identifications Database (PRIDE) under the accession number PXD015315 and analyzed using the Thermo Scientific Proteome Discoverer Software (Version 3.1.1.93). Additionally, proteomic data of resting and activated naive and memory CD4^+^ T cells published by Wolf et al. were re-analyzed [39]. The data-set is publicly accessible in the GEO with Accession Number GSE147229 and GSE146787 or via www.immunomics.ch (last visited 7th of December, 2023). Re-analysis was performed regarding protein abundance, protein renewal and protein degradation experiments. Graphs were generated using the annotation provided by the author.

### Database investigation to evaluate transcriptional CD74 gene regulation

Potential transcription factor binding sites at a maximum distance of 500 base pairs (bp) from the *CD74* gene locus were identified in the Gene Transcription Regulation Database (GTRD) http://gtrd2006.biouml.org/bioumlweb/#de=databases/EnsemblHuman85_38/Sequences/chromosomes%20GRCh38&pos=5:150400041-150514325, last visited on the 25th of May 2024) [40]. The PathwayNet database (https://pathwaynet.princeton.edu/predictions/gene/?network=human-transcriptional-regulation&gene=15273, last visited on the 25th of May 2024) and the STRING network analysis tool (https://string-db.org/cgi/network?taskId=bVkllE1RJOb3&sessionId=b4C13zpxyaPE, last visited on the 25th of May 2024) were used to identify relevant and MHC II-independent CD74 transcriptional regulation [41, 42].

### 3D migration of human peripheral blood-derived CD4^+^ T cells by time-lapse microscopy

The three-dimensional (3D) migration behavior of 72 h-activated human CD4^+^ T cells was assessed by time-lapse microscopy and individual cell tracking using the chemotaxis µ-Slide system from Ibidi GmbH (Munich, Germany). Briefly, CD4^+^ T cells (4 × 10^6^ cells) were seeded in rat tail collagen type I (Ibidi GmbH) gel in DMEM medium and subjected to a gradient of human MIF (concentration: 200 ng/ml) in the presence or absence of the neutralizing anti-CD74 antibody LN2 (sc-6262, Santa Cruz; 10 µg/ml) or the respective IgG control (sc-3877, 10 µg/ml) and the CXCR4 receptor inhibitor AMD3100 (A5602, Sigma Aldrich, 10 µg/ml). Cell motility was monitored performing time-lapse imaging every 1 min at 37 °C for 2 h using a Leica inverted DMi8 Life Cell Imaging System equipped with a DMC2900 Digital Microscope Camera with CMOS sensor and live cell imaging software (Leica Microsystems, Wetzlar, Germany). Images were imported as stacks to ImageJ software and analyzed with the manual tracking and chemotaxis and migration tool (Ibidi GmbH) plugin for ImageJ.

### Immunofluorescent staining

Cells were fixed with 4% paraformaldehyde (PFA) in PBS (Morphisto GmbH, Frankfurt a. M., Germany) for 15 min. For intracellular staining, cells were additionally permeabilized using TritonX-100 (Serva Electrophoresis, Heidelberg, Germany) in PBS for 10 min. After washing, T cells were blocked in 1% BSA in PBS for 1 h at RT. The blocking solution was removed and the cells incubated with primary antibodies against CD74 (LN2, sc-6262, 1:100, Santa Cruz), CXCR4 (PA3-305, 1:800, ThermoFisher), Bip (ab21685, 1:1000, abcam), or LAMP1 (H-228; 1:100, Santa Cruz) diluted in blocking buffer, at 4 °C overnight. After washing, secondary antibodies (goat anti-mouse Alexa-Fluor 647, A21235, Invitrogen; donkey anti-rabbit Cy3, 711–165-153, 1:300, Jackson ImmunoResearch) and, where indicated, 1 × DAPI was added to the sample and incubated in a humidity chamber for 1 h at RT. Samples were washed and prepared for microscopy using Vectashield^®^ mounting medium (Vector Laboratories, H-1000), either stored at 4 °C in the dark or analyzed directly using a LSM880 AiryScan confocal microscope (Carl Zeiss Microscopy GmbH, Jena, Germany).

### Proximity ligation assay (PLA)

For detection of CD74/CXCR4 protein complexes, 72 h-activated CD4^+^ T cells were stimulated with MIF in indicated concentrations for 40 min following fixation and PLA using the Duolink™ InSitu Orange Starter Kit Mouse/Rabbit (DUO92102) from Sigma Aldrich. For immunofluorescent staining and PLA, the Duolink^®^ PLA fluorescence protocol provided by the manufacturer was essentially followed, using primary antibodies against CD74 (sc-6262, 1:100, Santa Cruz) and CXCR4 (PA3-305, 1:800, ThermoFisher) as described above. Samples were then prepared for microscopy using Duolink^®^ mounting medium with DAPI, and coverslips sealed with commercially available nail polish and stored at − 20 °C until imaging on a Zeiss LSM880 AiryScan confocal microscope was performed. For quantification of complex formation, PLA dots per cell in four or more randomly selected fields of view were counted for each biological replicate.

### Statistical analysis

Statistical analysis was performed using GraphPad Prism Version 8.4.3 software. Unless stated otherwise, data are represented as means ± standard deviation (SD). After testing for normal distribution (evaluated using D’Agostino–Pearson testing or Shapiro–Wilk testing for small sample sizes and QQ plotting), data were analyzed either by two-tailed Student’s t-test or Wilcoxon matched-pairs signed-rank test, Mann–Whitney U test or unpaired t test with Welch's correction as appropriate. One-way ANOVA, Friedman test or Kruskal–Wallis test was performed, if more than two data sets were compared as appropriate. To account for multiple comparisons, either Dunnett’s or Dunn's multiple comparisons tests were applied as appropriate. Differences with *P* < 0.05 were considered to be statistically significant.

## Results

### Differentially regulated surface expression of MIF receptors CXCR4 and CD74 in primary human CD4^+^ T cells upon activation

**Fig. 1:**
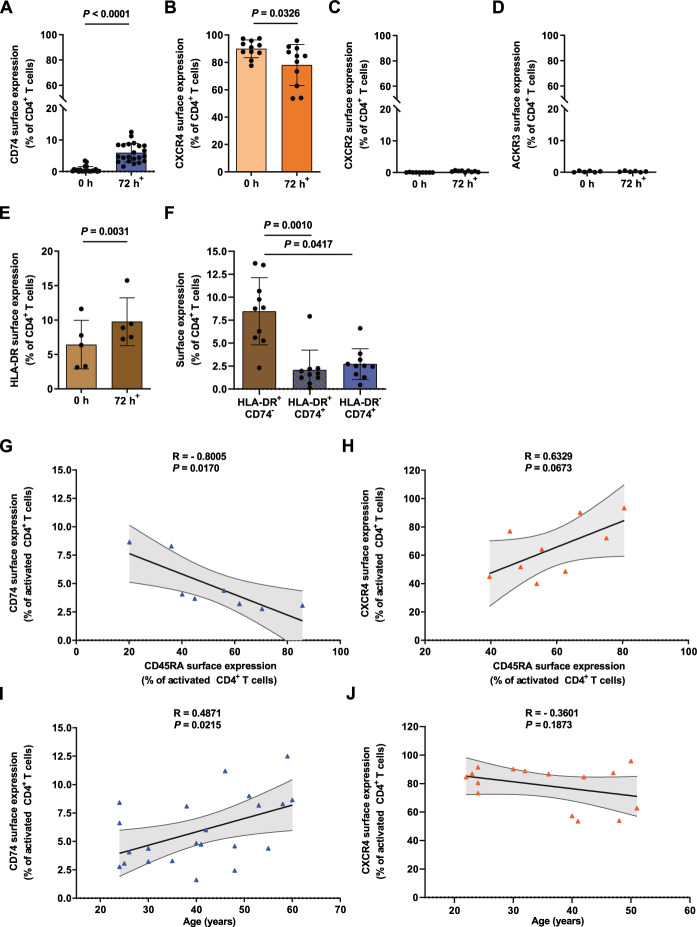
Cell surface MIF receptor profiling reveals inverse regulation of CD74 and CXCR4 upon T-cell activation. **A**–**D** MIF receptor profiling on primary human CD4^+^ T cells upon activation. Flow cytometry-based cell surface receptor profiling of the four MIF receptors CD74, CXCR4, CXCR2, and ACKR3, as indicated, on purified human CD4^+^ T cells before (0 h) and after 72 h of in vitro T-cell activation. Cell surface receptor-positive cells are plotted for each of the four receptors as percentage of CD4^+^ T cells. **E**, **F** MHC class II-independent expression of CD74 on activated CD4^+^ T cells. HLA-DR surface expression on CD4^+^ T cells before (0 h) and after 72 h of in vitro T-cell activation determined by flow cytometry*.* Comparison of percentages of HLA-DR^+^CD74^−^, HLA-DR^+^CD74^+^ and HLA-DR^−^CD74^+^ CD4^+^ T cells after 72 h of activation. For **A**–**F**, values are shown as means ± SD with individual datapoints representing independent donors (**A**, n = 22; **B**, n = 11; **C**, n = 9; **D**, n = 6; **E**, n = 5; **F**, n = 10). Differences between the 0 h and 72 h time points were analyzed by paired student’s t-test for **B**, **D**, **E**; by Wilcoxon matched-pairs signed-rank test for **A** and **C** and Friedman test with Dunn post-hoc test for **F** as appropriate. 72 h^+^ indicates time of in vitro T-cell activation in **A**–**E**. **G**, **H** Inverse correlation of CD74 and CXCR4 surface expression with the naive cell marker CD45RA. Correlation of surface CD74 and CXCR4 expression with the naive cell marker CD45RA in 72 h-activated CD4^+^ T cells as evaluated by flow cytometry. Data is displayed as scatter diagrams with individual data points shown (**G**, n = 8; **H**, n = 9). Pearson correlation coefficient was calculated for percentage of CD74^+^ and CXCR4^+^ vs. CD45RA^+^ cells. **I**, **J** Correlation between MIF receptor expression and donor age. Correlation between CD74 and CXCR4 surface expression and donor age after 72 h of T-cell activation. Data are depicted as scatter plots with individual data points shown (**I**, n = 22; **J**, n = 15). Pearson correlation coefficient was calculated for relation between the percentage of CD74^+^ and CXCR4^+^ T cells and donor age. For all panels statistical significance is indicated by actual *P* values.

In order to systematically investigate MIF receptor expression in the course of T-cell activation, we first performed a flow cytometry-based receptor profiling of the known MIF receptors CD74, CXCR4, CXCR2, and ACKR3 on freshly isolated primary human CD4^+^ T cells. The analysis confirmed an abundant expression of CXCR4 close to 90% in CD4^+^ T cells, whereas CD74, CXCR2, and ACKR3 showed no appreciable surface expression in non-activated CD4^+^ T cells (Fig. 1A–D, Supp. Fig. 2D–2G). [43, 44]. However, in vitro T-cell activation with anti-CD3/anti-CD28-coated beads for 72 h revealed a significant upregulation of CD74 surface expression from 0.65 ± 0.95 to 5.93 ± 2.97% (Fig. 1A), accompanied by a significant downregulation of CXCR4 from 89.85 ± 6.43 to 78.03 ± 15.03% (Fig. 1B). CXCR2 and ACKR3 surface expression levels remained unchanged upon activation (Fig. 1C, D, Supp. Fig. 2F and 2G).

The effectiveness of in vitro activation was verified by flow cytometry analysis of the surface activation markers CD45RA, indicating naive T cells, and CD45RO as a marker of activated effector and memory T cells, as well as for HLA-DR, a subunit of the MHC class II complex and previously described T-cell activation marker [45–49]. Activation led to a profound disappearance of the proportion of naive CD4^+^ T cells and shift towards the activated CD45RA^−^RO^+^ phenotype (Supp. Fig. 2A–2C). Consistent with previously published data, HLA-DR surface staining showed a significant activation-dependent increase in HLA-DR^+^CD4^+^ T cells from 6.43 ± 3.52 to 9.76 ± 3.47% after 72 h of activation (Fig. 1E). Co-analysis of both MHC-II related proteins CD74 and HLA-DR revealed that the majority of HLA-DR^+^ cells were CD74^−^. Focusing on the CD74^+^ population, we observed both HLA-DR^+^/CD74^+^ (2.07% ± 2.16%) double positive cells and a fraction of T cells (2.71% ± 1.68%) that expressed CD74 independent of MHC-II (Fig. 1F).

The observed inverse regulation of CD74 and CXCR4 upon activation was further confirmed by analyses revealing a close-to-significant positive correlation between CXCR4 and the naive T-cell marker CD45RA (r = 0.6329, P = 0.0673) and a significant negative correlation between CD74 and the naive cell marker CD45RA (r = − 0.8005, P = 0.0170) (Fig. 1G, H). Notably, correlation of CD74 and CXCR4 expression with donor age upon activation showed enhanced upregulation of CD74 (r = 0.4871, P = 0.0215), but only a non-significant trend towards a more pronounced downregulation of CXCR4 (r = − 0.3601, P = 0.1873) with increasing age (Fig. 1I, J).

### Abundant intracellular CD74 expression in resting CD4^+^ T cells and upregulation upon activation

**Fig. 2:**
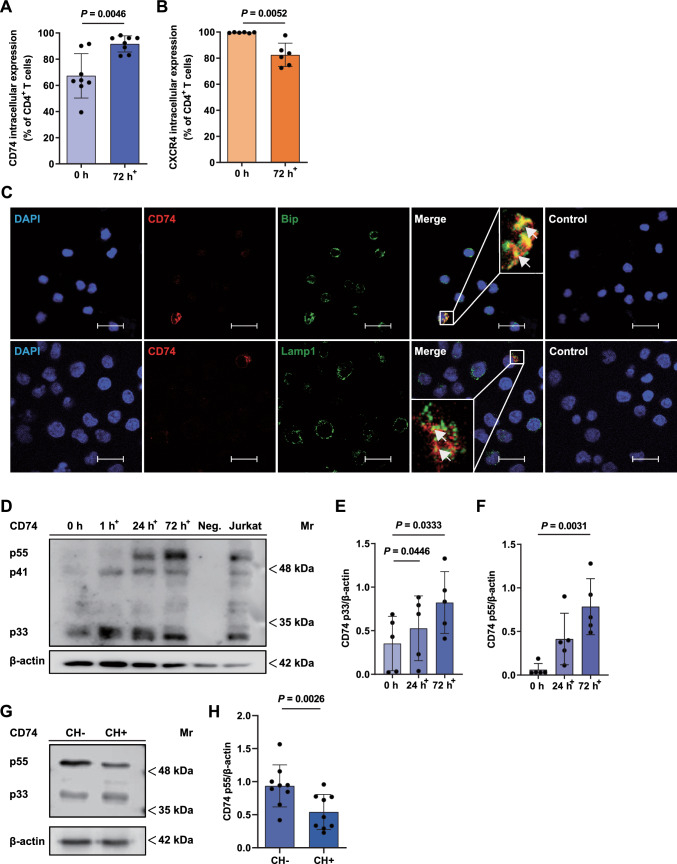
Constitutive expression and intracellular localization of CD74 in CD4^+^ T cells. **A**, **B** CD74 and CXCR4 expression in permeabilized CD4^+^ T cells before and after activation. Intracellular CD74 and CXCR4 expression was evaluated by flow cytometry of permeabilized freshly isolated (0 h) and 72 h-activated CD4^+^ T cells. Percentages of CD74^+^ (n = 8) and CXCR4^+^ (n = 6) cells are shown as means ± SD with individual datapoints representing independent donors. Statistical differences between the 0 h and 72 h time points were analyzed by paired student’s t-test. **C** Localization of CD74 in the endoplasmic reticulum (ER) and endolysosomal compartments. Immunofluorescent staining of CD74 (red) together with an ER (BiP, upper row, green) or lysosomal marker (LAMP1, bottom row, green) in 72 h in vitro activated and permeabilized CD4^+^ T cells imaged via CLSM (scale bar = 20 µm). Cell nuclei were counterstained with DAPI (blue). Samples stained with secondary antibodies alone served as controls. Arrows mark exemplary overlapping signals (yellow). Images shown are representative of three separate experiments. **D**–**F** CD74 protein expression in the course of CD4^+^ T-cell activation evaluated by SDS-PAGE/WB. CD4^+^ T cells were purified and lysed before (0 h) or after 1 h, 24 h or 72 h of in vitro T-cell activation following SDS-PAGE and WB analysis for CD74 and β-actin protein expression. Neutrophil cell lysates served as a negative control (Neg.), CD74 protein content of the Jurkat cell line was assessed without prior activation. OD values of the detected p33 and p55 CD74 isoforms before and after 24 h and 72 h of T-cell activation were determined and normalized to β-actin. Upregulation of the p33 and p55 isoforms is displayed as columns (means ± SD) with individual data points (n = 5). For comparison of 24 h and 72 h timepoints to 0 h control, statistical differences were analyzed by one-way ANOVA with Dunnett post-hoc test for **E** and Friedman test with Dunn post-hoc test for **F**. 1 h^+^, 24 h^+^, 72 h^+^ indicate the respective time of in vitro T-cell activation in **A**–**F**. **G**, **H** Evaluation of CD74 protein expression before and after chondroitinase treatment. 72 h-activated CD4^+^ T cells were lysed and treated with (CH+) or without (CH–) chondroitinase. SDS-PAGE and WB was performed as before for detection of CD74 and β-actin protein expression. Quantification of OD values of CD74 p55 in CH+  s. CH- samples normalized to β-actin displayed as bar chart (means ± SD) with individual data points (n = 9*)*. Statistical differences were analyzed by paired student’s t-test. For all bar diagrams, statistical significance is indicated by actual *P* values.

Only a small fraction of CD74 is known to be expressed on the cell surface, while most of CD74 is present in intracellular compartments. This prompted us to investigate intracellular CD74 and CXCR4 protein abundance in T cells via flow cytometry [50, 51]. Remarkably, in freshly isolated non-activated CD4^+^ T cells, we detected a high percentage of CD74^+^ cells (67.30% ± 16.94%) after membrane permeabilization pointing towards abundant CD74 protein expression even in resting conditions (Fig. 2A). Upon a 72 h-T-cell activation regime, we observed a significant further upregulation of CD74^+^ CD4^+^ T cells (67.30 ± 16.94 vs. 91.65 ± 6.178%) up to almost 100% (Fig. 2A). The initially observed variability of CD74 positivity most likely reflected individual donor characteristics, whereas in vitro T-cell activation aligned the T-cell populations leading to a more homogeneously increased percentage. Using the same experimental settings, the percentage of CXCR4^+^CD4^+^ T cells was determined before and after activation. CXCR4^+^CD4^+^ T cells were significantly diminished after 72 h activation from a baseline of nearly 100% in resting cells to approx. 85% (99.56% ± 0.3386% vs. 82.50% ± 8.965%) after activation. Nevertheless, CXCR4 remained abundantly expressed (Fig. 2B).

### Intracellular localization of CD74 within the ER and endolysosome

CD74 is typically located in cytoplasmic membranes such as the endoplasmic reticulum (ER), the Golgi apparatus and in endosomal or lysosomal vesicles [50, 51]. To verify a potential intracellular localization in these compartments, immunofluorescent co-staining of CD4^+^ T cells for CD74 together with the ER marker immunoglobulin binding protein (BiP) and the lysosomal marker lysosomal-associated membrane protein 1 (LAMP-1) were performed. Both the distribution pattern of CD74 signal surrounding the nucleus and the overlap of CD74 and BiP signals (yellow) indicate its presence primarily in the ER. Partial colocalization with LAMP-1 further suggests trafficking of CD74 within the endolysosomal compartment. Taken together, immunofluorescent staining of activated CD4^+^ T cells provided additional proof for CD74 expression and confirmed its localization within the cell in the ER/endolysosomal compartments (Fig. 2C).

### Upregulation of CD74 protein expression upon T-cell activation and identification of a chondroitin sulfate-modified p55 isomer

In order to verify and quantify CD74 protein expression in the course of T-cell activation, we performed additional time-dependent WB experiments from freshly isolated, 1 h-, 24 h- and 72 h-activated CD4^+^ T cells with an antibody against CD74. As expected, we observed protein bands at approx. 33 kDa and 41 kDa, corresponding to the most abundant human isoforms p33 and p41 (Fig. 2D) [52]. Quantification of CD74 protein expression was performed using the most reliably obtained p33 isoform and confirmed an upregulation of CD74 protein expression upon CD4^+^ T-cell activation (0 h: 0.35 ± 0.31 vs. 24 h: 0.53 ± 0.37 vs. 72 h: 0.82 ± 0.35) (Fig. 2E).

Surprisingly, further comparing non-activated and activated CD4^+^ T cells in the time-dependent WB experiments revealed an emerging protein band at 55 kDa (p55), which was only present after T-cell activation for 24 h and 72 h (0 h: 0.06 ± 0.07 vs 24 h: 0.41 ± 0.30 vs 72 h: 0.78 ± 0.32) (Fig. 2F). Lysates of Jurkat cells, an immortalized T cell clone that shares many of the features of primary human T cells, were electrophorized for comparison and contained not only the p33 and p41 isoforms, but also the novel p55 variant [53].

It seemed unlikely that p55 band signal is non-specific, as the band pattern was reproducible and was not observed in isolated primary human neutrophils that were included as a negative control in the experiment. The data are in line with previous reports of a specific post-translational chondroitinylated CD74 isoform, CD74-CS, running at about the same molecular weight [54–56]. Consistent with our observations on CD74 dynamics, previous studies showed a rapid and transient translocation of CD74-CS to the cell surface, followed by immediate endocytosis, so that only a small portion of CD74 was detected on the cell surface [54, 57–62]. Thus, the following experiment was designed to confirm the presence of a CD74-CS isoform. For this purpose, 72 h-activated CD4^+^ T cells were subjected to either PBS (CH–) or chondroitinase (CH+) treatment. Indeed, following chondroitinase treatment, we noticed the p55 signal intensity to be significantly decreased in comparison to non-treated controls pointing towards a rapid post-translational modification of CD74 with CS, which mediates CD74 translocation to the cell membrane (0.94 ± 0.32 vs. 0.54 ± 0.27*)* (Fig. 2G, H).

### In-depth confirmation of activation-dependent regulation of CD74 and CXCR4 by re-analysis of transcriptomic and proteomic data sets

**Fig. 3:**
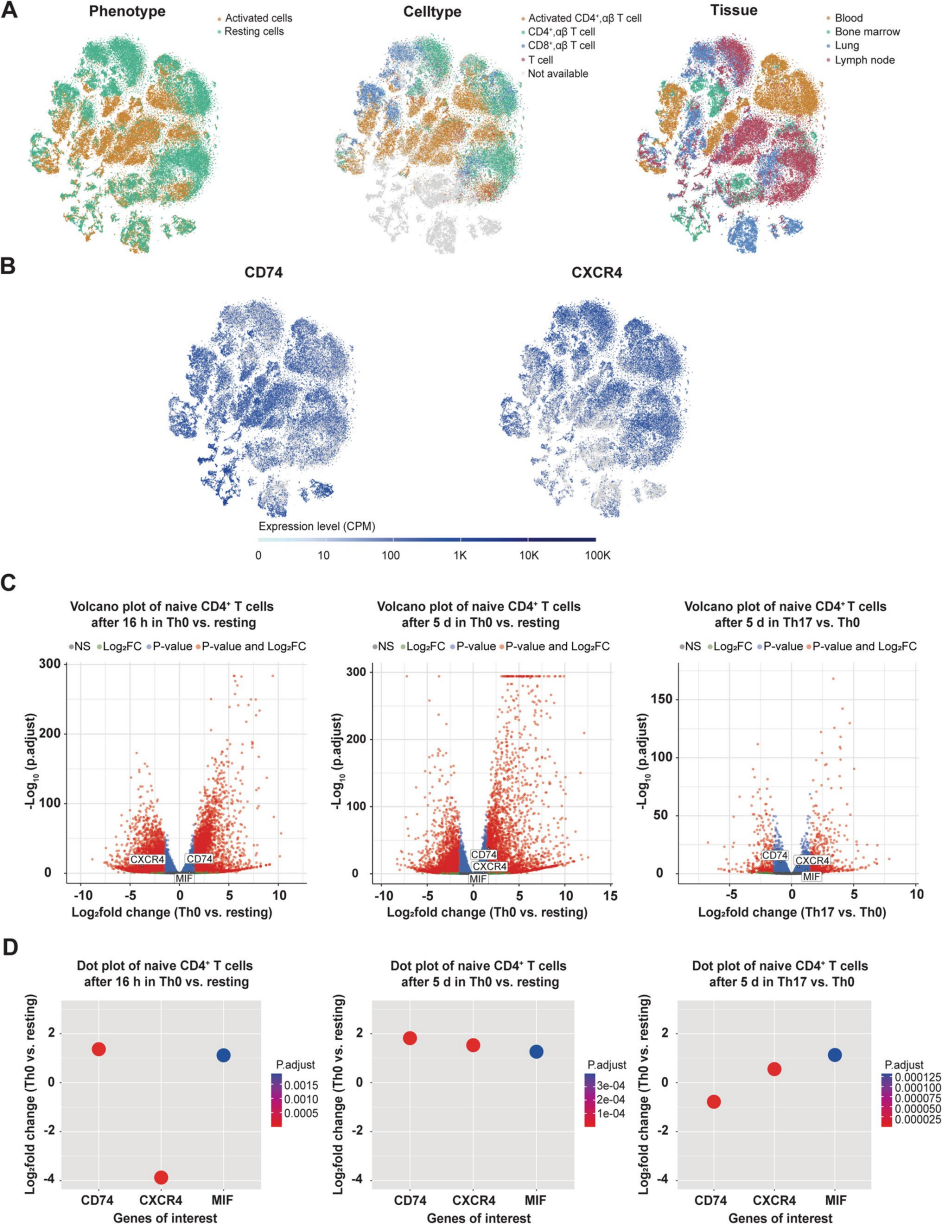
Evaluation of mRNA expression dynamics of CD74, CXCR4 and MIF in CD4^+^ T cells. **A**, **B** CD74 and CXCR4 mRNA expression in resting and activated CD4^+^ T cells. t-SNE embedding for the scRNAseq dataset obtained from Szabo et al. including scRNA data of CD3^+^ T cells from lung, lung draining lymph nodes and bone marrow of two deceased organ donors and PBMCs of two healthy volunteers [35]. Clusters depicted in the upper row colored by resting (green) vs. activated (orange) phenotype (left), by cell type (middle, orange: activated CD4^+^ αβ T cells, green: CD4^+^ αβ T cells, blue: CD8^+^ αβ T cells, red: T cells, gray: not available) or by tissue (right, orange: blood, green: bone marrow, blue: lung, red. lymph node). mRNA expression levels are depicted in copies per million (CPM) reads of CD74 and CXCR4. **C**, **D** DGE analysis of CD74, CXCR4 and MIF depending on T-cell activation and cytokine polarization in naive CD4^+^ T cells. Re-analysis of publicly available bulk-RNAseq data of naive CD4^+^ T cells from three healthy individuals in different activation and cytokine polarization conditions by Cano-Gamez et al. regarding DGE analysis of CD74, CXCR4 and MIF highlighted in volcano plots (upper row, red: genes with log2fold >|1,5| and adjusted *P* < 0.05 changes, blue: genes with log2fold <|1,5| and adjusted *P* < 0.05 changes, green: genes with log2fold >|1,5| but non-significant (ns) changes, grey: genes with log2fold <|1,5| and ns changes) and in dot blots (bottom row, dots highlight significant results between experimental groups with adjusted *P* < 0.05, color scale indicates the respective p-values) including comparison of Th0 (activated without cytokine polarization) vs. resting (non-activated controls) conditions after 16 h (left) and 5 d (middle) as well as Th0 vs. Th17 cytokine polarization after 5 d (right) [28].

To gain a deeper insight into the regulation of CD74 in CD4^+^ T cells, we re-analyzed publicly available scRNA-seq data from Szabo et al. [35]. scRNA data was retrieved from data sets of resting and CD3/CD28-activated (16 h) blood, lung, lymph node and bone marrow-derived CD3^+^ T cells from two deceased adult organ donors and PBMCs of two healthy blood donors. Ubiquitous expression of CD74 and CXCR4 was clearly evident in both activated and resting T-cell phenotypes (Fig. 3A). However, enhanced expression of CD74 was detected mainly in activated T-cell clusters, whereas enhanced CXCR4 expression was mainly observed in cells with a resting phenotype. Of note, a comparable inverse activation pattern for CD74 and CXCR4 was noted in CD8^+^ T cells (Fig. 3B).

To verify these results and to assess whether CD74, CXCR4 and MIF expression is influenced by cytokine conditions driving CD4^+^ T-cell differentiation towards T-cell effector phenotypes during CD3/CD28 activation, we further re-analyzed a publicly available data set of Cano-Gamez et al., who performed a bulk-RNAseq analysis of polarized (resting: no activation, no added cytokines; Th0: control with no added cytokines; Th1: IL-12, anti-human IL-4 antibody; TH2: IL-4, anti-human IFN-γ antibody, Th17: IL-6, IL-23, IL-1β, TGF-β1, anti-human IL-4 antibody, anti-human IFN-γ antibody; iTreg: TGF-β1, IL-2; IFN-β-stimulated group) naive CD4^+^ T cells after 16 h and 5 d of stimulation (Fig. 3C, D) [28]. DEG analysis confirmed a significant upregulation of CD74 (log2fold change 16 h: 1.37; 5 d: 1.82) and MIF (log2fold change 16 h: 1.12; 5 d: 1.27) expression in 16 h- and 5 d-activated naive T cells, when comparing the resting and Th0 experimental groups. CXCR4 expression in turn was significantly downregulated after 16 h, but showed enhanced expression after 5 d of activation in Th0 vs. resting naive T cells (log2fold change 16 h: − 3.87; 5 d: 1.53). To analyze cytokine-induced polarization of T cells, we performed DEG analysis of 16 h- and 5 d-activated naive T cells (Th0) with the respective polarized experimental group. In fact, most of the cytokine conditions did not lead to any significant changes in CD74, CXCR4 or MIF expression. The only observed significant change regarding CD74 expression was a downregulation in Th17 cells at 5 d (log2foldchange: − 0.77986), accompanied by an upregulation of CXCR4 (log2foldchange: 0.553153) and MIF (log2foldchange: 1.130575). Overall, CD74 mRNA expression was markedly upregulated by T-cell activation in naive CD4^+^ T cells, while the specific cytokine milieu only showed minor effects. Inverse regulation of the MIF receptors CD74 and CXCR4 during the early activation process was confirmed on mRNA level. Additionally, obtained data provides further evidence of an increased MIF expression upon T-cell activation (Fig. 3D). To assess whether these smaller effects of additional cytokine polarization on CD74 and CXCR4 mRNA levels are also reflected on protein level, we re-analyzed the proteomic data of 5 d-polarized CD4^+^ memory T cells from the Cano-Gamez et al. study [28]. Re-analysis confirmed an upregulation of CD74 protein upon T-cell activation, whereas cytokine polarization to T-cell phenotypes did not have any significant impact on CD74 protein abundance (Supp. Fig. 3A). In contrast, CXCR4 protein abundance was markedly increased upon cytokine-driven polarization towards Treg and Th17 phenotypes (Supp. Fig. 3B).

**Fig. 4:**
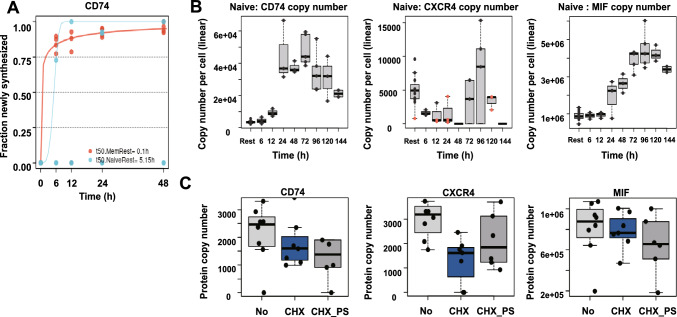
Evaluation of protein dynamics of CD74, CXCR4 and MIF in CD4^+^ T cells. **A** Rapid renewal of CD74 in resting memory CD4^+^ T cells. CD74 protein renewal rates in naive (blue) vs. memory (orange) CD4^+^ T cells. Fraction of newly synthesized protein calculated from LC–MS/MS analysis of pulsed SILAC of resting CD4^+^ T cells. Analysis conducted after 0, 6, 12, 24 and 48 h in culture. n = 3–4. **B** Time course of CD74, CXCR4 and MIF protein expression upon activation in naive CD4^+^ T cells. CD74 (left), CXCR4 (middle) and MIF (right) copy number per cell in naive CD4^+^ T cells. Label-free quantification of proteins via the MaxQuant algorithm without and after 6, 12, 24, 48, 72, 96, 120 and 144 h of in vitro activation. Proteins identified by MS/MS (black dots) or matching (orange dots). Estimation of copy number per cell based on protein mass of cell. n = 7 for resting naive T cells; n = 3 for 6 h, 12, 48 h, 120 h T cells, n = 4 for 24 h, 72 h, 96 h activated T cells. **C** Analysis of protein degradation in naive CD4^+^ T cells. Protein copy numbers of CD74 (left), CXCR4 (middle) and MIF (right) in naive CD4^+^ T cells without treatment (No), with 24 h of cycloheximide treatment alone (CHX, 50 μg/ml) or in combination with 10 μM bortezomib (CHX_PS). Box plots depict median and interquartile range (IQR). Whiskers show lowest data point contained in the 1.5 IQR of lowest quartile and highest data point contained in the 1.5 IQR of highest quartile. n = 5 for No, n = 4 for CHX and n = 6 for CHX_PS. Data in **A**–**C** retrieved from Wolf et al. [39].

Next, we re-analyzed the proteomic data set of Wolf et al., who studied mRNA translation kinetics, protein turnover and synthesis rates in human naive and activated T cells, to gain a better understanding on the dynamics of CD74 protein expression in CD4^+^ T cells [39]. At first, we assessed the data on protein turnover and renewal under resting conditions. For this experiment, Wolf et al. measured protein synthesis and turnover rates of non-activated naive and memory CD4^+^ T cells by applying stable isotope labeling of amino acids in cell culture (SILAC) and subsequent liquid-chromatography coupled mass spectrometry (LC–MS/MS) analysis. The protein synthesis rate was determined based on the proportion of newly synthesized, heavy isotope-labeled amino acid-containing proteins to total protein content after 6, 12, 24 and 48 h of cultivation. The study identified ETS1, a proto-oncogene associated with survival, activation and proliferation in T cells as the most rapidly renewed transcription factor (renewal ratio of 0.99 after 24 h, estimated half-life of less than 1 h) [63, 64] (Supp. Fig. 3C). Of note, the retrievable data on CD74 renewal yielded comparable results (protein renewal ratio of 0.92 after 24 h, estimated half-life less than 1 h) and thus revealed that CD74 is among the proteins with fastest renewal and turnover rates in resting memory T cells (Fig. 4A). This effect was much less pronounced in naive T cells with a renewal ratio below 50% after 24 h, possibly linking CD74 to homeostasis and preparedness of memory T cells. Supp. Fig. 3C and 3D show protein renewal rates of selected other proteins for further comparison. Re-analysis of protein abundance in naive CD4^+^ T cells in the course of CD3/CD28 activation confirmed our previous findings showing an upregulation of CD74 and downregulation of CXCR4 protein levels upon activation (Fig. 4B). CD74 upregulation began at 12 h with peak expression of CD74 protein observed after 72 h of activation both in naive and memory T cells with an observed timespan of upregulation of up to 120 h. For comparison, we analyzed the proteomic time course of CD69, IL2Rα/CD25 and HLA-DR, i.e. well-established T-cell activation markers. CD74 upregulation occured between the ‘early’ marker CD69 and the ‘intermediate’ activation marker CD25 (Supp. Fig. 3E–3G) [65, 66]. The dynamics of CXCR4 protein expression in naive T cells confirmed the previously observed inverse profile and indicated an immediate down-regulation of CXCR4 protein with a minimum protein abundance seen after 48 h of activation with following protein reconstitution towards 96 h, supporting our above mentioned finding of initially downregulated and later-on induced mRNA expression. We also analyzed MIF in these data sets. Similar to the upregulation pattern seen for CD74, MIF protein was also markedly enhanced upon activation and showed elevated expression in resting naive and memory T cells starting from 24 h, with a peak observed at 96 h (Fig. 4B). In order to specifically address protein degradation, Wolf et al. quantified protein copy numbers by LC–MS/MS in naive CD4^+^ T cells after inhibition of mRNA translation by cycloheximide (CHX) alone or in combination with bortezomib (PS), a specific inhibitor of the 26S proteasome. CD74 protein levels were only mildly affected by blockade of protein synthesis, speaking in favor of a low protein degradation rate and consistent with a lower renewal in resting naive CD4^+^ T cells. As CD74 was previously described to be degraded strictly sequentially in the endolysosomal system, additional treatment with PS confirmed the expected proteasome-independent degradation of CD74, while CXCR4 is most likely partially degraded via the proteasome (Fig. 4C) [67]. Furthermore, inhibition of proteasomal degradation did not recover MIF protein levels, suggesting a proteasome-independent degradation of MIF in resting T cells (Fig. 4C).

### Exploring MHC II-independent CD74 transcriptional gene regulation

To explore potential MHCII-independent CD74 transcriptional gene regulation, we performed a database analysis using the Gene Transcription Regulation Database (GTRD) yielding 375 different transcription factor binding sites within a maximum distance of 500 bp from the *CD74* gene locus (Supp. Table 2) [40]. Relevant results were narrowed down by predicting the genes involved in the transcriptional regulation of CD74 using the PathwayNet database [41]. Genes with a relationship confidence of more than 0.1 were included for further consideration (Supp. Table 3). Of the 19 transcription factors identified, four lacked a binding site within 500 bp of the CD74 gene and were therefore excluded. Furthermore, STRING network analysis identified the seven transcription factors with the highest relationship confidence as MHC II transactivator (CIITA)-associated genes, representing the master regulator of MHC II class gene expression (Supp. Fig. 4) [42, 68]. Assuming a common transcriptional regulation of MHC II proteins and CD74 by these transcription factors, we excluded these hits from our search as well [68, 69]. Among the remaining eight transcription factors, ETS1, a proto-oncogene associated with survival, activation and proliferation in T cells, seemed particularly noteworthy, as it was only recently identified by Wolf et al. as the most rapidly renewed transcription factor in T cells reflecting preparedness towards activating stimuli [39, 63, 64]. By performing an assay for transposase-accessible chromatin (ATAC) and ChIP sequencing, Wolf et al. further investigated genes regulated by ETS1 in CD4^+^ T-cells [39]. Revisiting the ATAC and ChIP supplemental material of that study, we identified the *CD74* gene to be located in ETS-1-accessible chromatin regions in resting naive CD4^+^ T cells and revealed actual ETS1 binding in the CD74 promoter region, both suggesting an ETS1 transcriptional regulation of *CD74* in CD4^+^ T cells. Binding of ETS1 to other MHC II-associated genes was not observed. In conclusion, these data reflect an independent regulation of gene expression for CD74 and MHC II in resting naive CD4^+^ T cells and identify ETS1 as an associated transcription factor.

### Involvement of CD74 and CXCR4 in MIF-mediated CD4^+^ T-cell chemotaxis

**Fig. 5:**
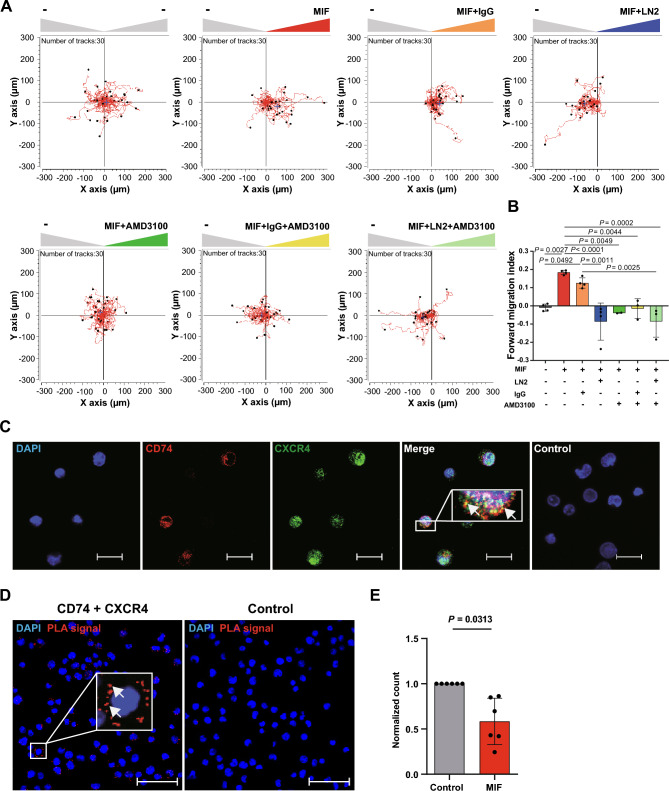
Involvement of CD74 and CXCR4 in MIF-mediated CD4^+^ T-cell chemotaxis. **A** Both MIF receptors CXCR4 and CD74 are required for MIF-elicited migration of activated CD4^+^ T cells as assessed by 3D chemotaxis assay. Representative trajectory plots (x, y = 0 at time 0 h) of migrated activated CD4^+^ T cells (72 h) in a three-dimensional (3D) aqueous collagen-gel matrix towards a MIF chemoattractant gradient (MIF concentration: 200 ng/ml, –: control medium) that was established in presence or absence of a CD74 neutralizing antibody, a corresponding isotype control (IgG) or the CXCR4 receptor inhibitor AMD3100. Cell motility was monitored by time-lapse microscopy for 2 h at 37 °C, images were obtained every minute using the Leica DMi8 microscope. Single cell tracking was performed of 30 cells per experimental group. The blue crosshair indicates the cell population’s center of mass after migration. **B** Quantification of the 3D chemotaxis experiment in **A** showing inhibition of MIF-induced CD4^+^ T-cell migration upon co-incubation with CD74 neutralizing antibody and AMD3100 either alone or in combination. Plotted is the calculated forward migration index (FMI, means ± SD) based on manual tracking of at least 30 individual cells per treatment (n = 2–4). Statistical differences were analyzed by one-way ANOVA with Tukey post-hoc test and indicated by actual *P* values. **C** Cell surface colocalization of the MIF receptors CD74 and CXCR4 on activated CD4^+^ T cells. Immunofluorescent cell surface staining of CD74 (red) and CXCR4 (green) either alone or in combination on 72 h-activated CD4^+^ T cells imaged via CLSM (scale bar = 20 µm). Cell nuclei were counterstained with DAPI (blue). Samples stained with secondary antibodies alone served as controls. Images shown are representative of two independent experiments. **D**, **E** Proximity ligation assay indicating CD74/CXCR4 heterocomplex formation and MIF dependent internalization. **D** Display of a representative PLA result visualizing the interaction of CD74 and CXCR4 on the cell surface of 72 h-activated CD4^+^ T cells (red dots indicating positive PLA signal; imaged via CLSM; 40 × objective, DAPI, blue; scale bar: 50 µm). **E** Quantification of CD74/CXCR4 heterocomplexes on the cell surface of 72 h-activated CD4^+^ T cells upon stimulation with MIF (200 mg/ml) prior to fixation (means ± SD of PLA dots /cell normalized to control, n = 6). Statistical differences were analyzed by Wilcoxon matched-pairs signed-rank test and indicated by actual *P* values

One key attribute of T cells is their ability to migrate towards sites of inflammation. MIF-mediated T-cell recruitment is a well characterized atherogenic MIF effect that has been assumed to be primarily mediated via CXCR4 [4, 70]. In order to determine the functional relevance of CD74 surface upregulation in activated human CD4^+^ T cells, we assessed their migratory capacity in response to MIF applying a 3D chemotaxis assay that allows for tracking single cell migration trajectories via live cell imaging. MIF potently promoted chemotactic migration of activated CD4^+^ T cells in a bell-shaped dose–response behavior typically observed for chemokines, with maximal MIF-induced chemotaxis seen at 200 ng/ml of MIF (Supp. Fig. 5A and 5B). Therefore, this concentration was used for all subsequent migration assays. In a next step, we performed co-incubation experiments with AMD3100, a selective pharmacological CXCR4 inhibitor and the CD74-neutralizing antibody LN2. MIF-induced chemotaxis was fully abrogated when MIF was co-incubated with AMD3100 and LN2 either alone or in combination, while incubation of T cells with the inhibitors alone or isotype control immunoglobulin (IgG) showed no significant effects on cell motility (Fig. 5A, B, Supp. 5C and 5D). Taken together, we show involvement of CD74 and CXCR4 in MIF-elicited chemotaxis of activated CD4^+^ T cells. Mechanistically, joint involvement of CD74 and CXCR4 may be explained by CD74/CXCR4 heterocomplex formation as previously observed in model cell lines after overexpression or by synergistic/converging signaling pathways [8].

### CD74 and CXCR4 complex formation in activated CD4^+^ T cells determined by proximity ligation assay

To evaluate whether CD74 and CXCR4 heterocomplex formation occurs in activated CD4^+^ T cells, we first established immunofluorescent co-staining of CD74 and CXCR4 on 72 h-activated CD4^+^ T cells. Stainings were performed without cell permeabilization to specifically detect cell surface-bound receptors. Widefield and confocal laser scanning microscopy (CLSM) provided initial evidence for a colocalization of CD74 and CXCR4 on 72 h-activated T cells (Fig. 5C). To investigate whether colocalized CD74 and CXCR4 indeed form heterocomplexes, a PLA was performed which detects inter-molecular interactions within a distance of < 40 nm and represents an established method to identify chemokine receptor heterocomplexes [12]. Specific PLA signals were detected in 72 h-activated T cells, demonstrating the occurrence of CD74 and CXCR4 heterocomplexes (Fig. 5D). Stimulation with 200 ng/ml MIF significantly decreased PLA-signal indicating a MIF-induced signal transduction by internalization of CD74/CXCR4 receptor complexes (Fig. 5E). To our knowledge these results provide the first evidence of CD74/CXCR4 heterocomplex internalization in the context of MIF signaling.

### CD74 surface upregulation in CD4^+^ and CD8^+^ T cells during severe COVID-19 infection

**Fig. 6:**
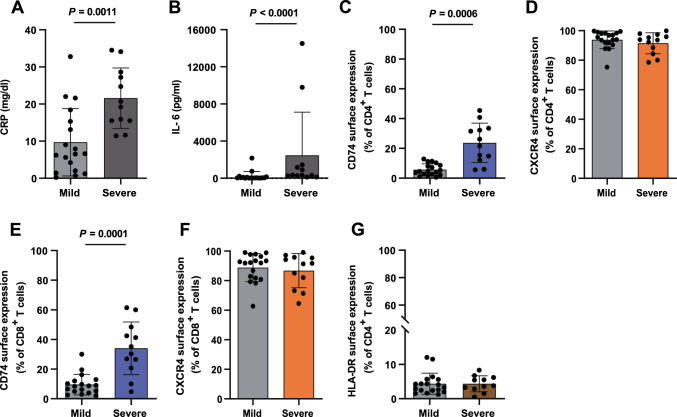
MHC-II independent upregulation of CD74 in T cells of critically ill COVID-19 patients. **A**, **B** Increased inflammatory markers CRP and IL-6 in patients with severe COVID-19 disease. Serum peak concentrations of inflammatory markers CRP (mg/dl) and IL-6 (pg/ml) from laboratory results of patients with mild (WHO 1–3, n = 18) vs. severe (WHO ≥ 5, n = 12) COVID-19 disease. **C**–**F** CD74 and CXCR4 surface expression on CD4^+^ and CD8^+^ T cells from mild vs. severe disease patients. **G** No significant differences in HLA-DR surface expression in COVID-19 patient cohorts classified by disease severity. Results of a flow cytometry-based cell surface receptor profiling. Bar charts in **A**–**G** show means ± SD with individual datapoints representing independent patients. Cell surface receptor-positive cells are plotted as percentages of the respective T-cell phenotype. Statistical differences were analyzed by unpaired t test for **A** and **C** and Mann–Whitney U test for **B**, **D**, **E**, **F**, and **G** and indicated by actual *P* values.

Finally, to explore the translational relevance of our findings, we assessed CD74 and CXCR4 surface expression in T cells and monocytes isolated from patients with mild (WHO 1–3) and severe (WHO grade ≥ 5) COVID-19 disease, which were obtained from the COVID-19 Registry of the LMU University Hospital Munich (CORKUM). Due to the retrospective approach of this study and heterogeneity of available time points for each patient, we chose to evaluate the MIF receptor profile at time points closest to admission to the hospital. As not all laboratory indices were available at any given time point, we identified the inflammation peak for each patient defined as the highest measured CRP or IL-6 value for additional comparison of both groups. As expected, the inflammation markers CRP (9.71 ± 9.07 vs. 21.58 ± 8.15) and IL-6 (215.1 ± 516.5 vs. 2464 ± 4654) were significantly increased in the severely affected patients (Fig. 6A).

In line with a recently published report by Westmeier et al., we observed a significant upregulation of CD74 surface expression on CD4^+^ (5.71% ± 3.87% vs. 23.75% ± 13.24%) and CD8^+^ (9.52% ± 6.95% vs. 34.02% ± 17.80%) T cells in the severe disease group compared to patients with mild disease (Fig. 6C, E) [71]. Notably, CD74 expression was higher in the CD8^+^ T cells (34.02% ± 17.80%) compared to CD4^+^ T cells (23.75% ± 13.24%) among severe patients. In contrast, we observed no significant differences between both groups regarding CXCR4 and HLA-DR surface expression again pointing towards an HLA-DR-independent upregulation of CD74 (Fig. 6D, F, G). When comparing CD74 and CXCR4 surface expression on monocyte populations, we further observed a significant upregulation of CD74 in classical (CD14^++^CD16^−^) monocytes in the severe disease group compared to patients with mild disease (Supp. Fig. 6A–6E). Overall, we confirmed an upregulation of the MIF receptor CD74 in CD4^+^ and CD8^+^ T cells in critically ill COVID-19 patients.

## Discussion

Here, we provide novel insights in constitutive and activation-dependent mRNA and protein dynamics of CD74 in CD4^+^ T cells. Our analyses reveal CD74 upregulation, post-translational modification with CS and MHC II-independent translocation to the cell surface upon T-cell activation. Surface CD74 forms heterocomplexes with the classical chemokine receptor CXCR4 and is mechanistically involved in MIF-elicited T-cell chemotaxis. Dysregulated CD74 expression in severe COVID-19 disease patients demonstrates the translational relevance of our findings.

Most likely due to its classical and well-established MHC II-related functions, CD74 was initially overwhelmingly studied in antigen-presenting cells, most notably monocytes/macrophages and B cells [1]. The discovery of CD74 as the cognate MIF receptor has partially changed this picture. In the course of these studies, MIF/CD74 pathways were not only examined in monocytes and macrophages, but it turned out that CD74 can be abundantly expressed in several types of cancer cells and may be upregulated in certain other cell types such as endothelial cells or cardiomyocytes upon inflammatory stimulation or stress [21, 26–28]. However, MHC class II-negative T cells have mostly been neglected in this regard. Only a handful of descriptive reports on CD74 expression in human T cells exist, mainly in context of disease, and without scrutinizing any mechanisms. Yang et al. investigated CD74 surface expression in PBMCs after stroke and amongst other cell types found a significant increase in the number of CD74-expressing CD4^+^ T cells but not CD8^+^ T cells [26]. Fagone et al. showed an upregulation of CD74 gene expression in CD4^+^ T cells upon activation, that was unchanged in T cells from healthy donors vs. patients with multiple sclerosis [27]. In contrast, in the chronic inflammatory context of rheumatoid arthritis, Sánchez-Zuno et al. observed the percentage of CD74 expressing T cells to be below 1% [72]. To our knowledge, Gaber et al. provided the only functional evidence of CD74 in human CD4^+^ T cells reporting on an inhibition of MIF-induced T-cell proliferation using a neutralizing CD74 antibody [21]. However, the relevance of this observation has remained unclear, as no isotype control immunoglobulin was used in that study. In contrast to CD74, regulation of CXCR4 in T cells has been studied comprehensively, also as it plays an important role in the docking-process of the human immunodeficiency virus and mediates CXCL12-driven co-stimulatory and migratory T cell responses [73–76].

Our MIF receptor profiling of freshly isolated primary human CD4^+^ T cells revealed the expected abundant expression of CXCR4, whereas no substantial surface expression of CD74, CXCR2 and ACKR3 could be detected. This identifies non-activated human CD4^+^ T cells as a suitable cell type to study the MIF/CXCR4 axis. In previous reports, CXCR4 expression was shown to be downregulated in the context of T-cell activation, which is confirmed by our study [73, 75]. Nevertheless, CXCR4 remained abundantly expressed also in activated T cells.

An unanticipated effect was the observation of a significant upregulation of CD74 surface expression upon T-cell activation. Of note, this upregulation was independent of HLA-DR pointing towards an MHC II-independent role of CD74 in CD4^+^ T cells. Interestingly, CD74 surface expression correlated with donor age, indicating a potentially more pronounced CD74 upregulation in memory and effector T cells compared to naive T cells, due to physiologically increased abundance of these phenotypes upon enhanced antigen encounters during aging [77–79].

Our MIF receptor profiling of resting and activated CD4^+^ T cells as well as re-analysis of CD4^+^ T-cell proteome data from Wolf et al. revealed no expression of CXCR2 in T cells, which is in line with multiple literature reports, but stands in contrast to the recent finding of CXCR2/CD74 co-expression in T cells as reported by Westmeier et al. [80]. Expression of ACKR3 in T cells still remains controversial [81, 82].

As CD74 is known to be expressed only in small percentages on cell surfaces and is mainly stored in intracellular deposits, we next evaluated CD74 protein expression after membrane permeabilization via flow cytometry. Unexpectedly and to date unknown, we detected an abundant intracellular expression of CD74 in freshly isolated T cells, which was further enhanced by T-cell activation. WB experiments confirmed enhanced CD74 expression with detection of protein bands corresponding to the known p33 and p41 isoforms in humans [1, 52, 83, 84]. However, due to the small difference in size a clear differentiation between short and long isoforms of the protein regarding p33 vs. p35 and p41 vs. p43 isoforms was not possible. Interestingly, we observed an additional pronounced protein band at approximately 55 kDa, which appeared only after 24 h of T-cell activation and further increased in abundance during activation, even exceeding the most abundant p33 protein band. Previous reports identified a specific CD74 isoform, CD74–CS that is being reported to run at a similar molecular weight and is product of a post-translational modification with the glycosaminoglycan CS at Ser 201. The modification was shown to enable the translocation of CD74 molecules towards the cell surface, while due to following rapid endocytosis only a small proportion can be transiently detected on the cell surface [54–62]. In fact, when we treated our T-cell samples with chondroitinase, an enzyme that specifically cleaves CS, we noticed the signal intensity of the observed p55 isoform to be significantly decreased in comparison to untreated controls. Nevertheless, we acknowledge that treatment with chondroitinase did not lead to a complete disappearance of the observed band, which could be explained by sub-optimal buffer conditions due to the strong pH-dependency of the enzyme or non-sufficient incubation time. Furthermore, several other post-translational modifications, such as O- and N-silylation, palmitoylation and phosphorylation, have been reported for CD74 that were not studied in this work [85–87]. Despite these limitations, we speculate that post-translational modification of CD74 with CS might be the underlying mechanism of CD74 translocation to the cell surface during the process of T-cell activation. Immunofluorescent co-staining of CD74 with ER and lysosomal markers verified the typical localization of CD74 in the ER and suggested a functional trafficking of CD74 within the endolysosomal compartment. Re-analysis of two independent RNAseq data sets from the Cano-Gamez et al. and Szabo et al. studies and two proteomic data sets from the Cano-Gamez et al. and Wolf et al. publications comparing resting and activated T-cell states, complemented our data and provided substantial corroborating evidence that CD74 is constitutively expressed in resting T cells and becomes rapidly upregulated upon T-cell activation in a sustained manner [28, 35, 39]. The proteome data suggested a maximum CD74 protein abundance after 72 h and again identified a counter-regulation of CD74 and CXCR4 in the early activation phase. After the initial downregulation, CXCR4 expression was then found to be reconstituted after approximately 3 to 4 d. Of note, CD74 upregulation occurred after upregulation of the early activation marker CD69, but before the intermediate activation marker CD25 [65, 66]. Cytokine polarization to T-cell effector phenotypes had no additional effects on CD74 protein abundance. In contrast, CXCR4 protein expression was upregulated after 5 days of Treg and Th17 polarization, possibly linked to an already described TGF-β-induced CXCR4 expression mechanism [88].

The study by Cano-Gamez et al. caught our attention as CD74 incidentally appeared as a strong marker protein of natural Tregs and effector memory T cells re-expressing CD45RA (TEMRA) in their presented data, possibly linking CD74 protein expression to T-cell effectorness [28]. Since observations of CD74 expression have often been made under inflammatory conditions, as for instance IFN-γ-rich environments, or in a disease context, we compared DEGs of regularly activated T cells (Th0) with activated T cells that were additionally differentiated towards Th0, Th1, Th2, iTreg and Th17 phenotypes through established cytokine polarization protocols [89]. Notably, except for the observed reduction of CD74 in Th17 conditions, cytokine conditions did not trigger significant changes. Therefore, T-cell activation represents the main stimulus for CD74 upregulation independent of the surrounding inflammatory cytokine milieu. Interestingly, Th17-polarized cells were also the only phenotype with significantly upregulated MIF expression compared to non-polarized CD4^+^ T cells, fitting to previous data indicating a role of MIF in Th17 T-cell differentiation [18, 20, 24]. Re-analysis of proteomic data further identified CD74 to be rapidly renewed in resting memory CD4^+^ T cells, potentially pointing towards a role of CD74 in memory T-cell homeostasis.

We also aimed to identify potential MHC II-independent *CD74* transcriptional gene regulation. Combining a database analysis of the GTRD, PathwayNet and STRING network databases enabled us to narrow down relevant and potential MHC II-independent transcription factors within a 500 bp distance from the CD74 gene locus. However, we like to emphasize that the here provided database research approach mainly relies on the quality of the included pathway/protein interaction prediction tools and can only be interpreted as a first approximation to the subject. The list of eight CIITA-independent transcription factors with high confidence predictions included ETS1, a crucial transcription factor for T-cell survival and activation [63, 64]. In this context, Wolf et al. identified ETS1 as the most rapidly renewed transcription factor in T cells reflecting preparedness towards activating stimuli [39]. Accordingly, by performing an ATAC assay, Wolf et al. found that the ETS1 transcription factor binding motif can be detected in accessible promoter regions of the resting naive CD4^+^ T-cell genome. About half of these binding sites were located in promoter regions, suggesting ETS1 as a transcriptional regulator of the promoter-associated genes. Interestingly, supplementary data of Wolf et al. shows that the CD74 gene is located in accessible chromatin regions in naive CD4^+^ T cells. Based on a ChIP analysis, showing actual ETS1 binding in the CD74 promoter region, transcriptional regulation of CD74 by ETS1, a transcription factor associated with T-cell preparedness for rapid activation, seems conceivable. Binding of ETS1 to other MHC II-associated genes was not observed, which may be either related to insufficient accessibility of the MHC II-related genes in resting naive CD4^+^ T cells or differential ETS1 gene binding.

Taken together, we hypothesize that ETS1-driven regulation of CD74 expression might be the underlying process of the observed rapid CD74 induction after activation, which, together with post-translational chondroitin sulfatinylation of constitutively expressed intracellular CD74, serves to rapidly establish marked CD74 surface expression. Once positioned on the cell surface, CD74, functioning as the cognate MIF receptor, can mediate downstream signaling events [90].

In the absence of an identified classical signaling-competent cytosolic domain in the short cytoplasmic tail of CD74, two alternative distinct tracks of CD74 signaling have been reported. First, CD74 signaling can be mediated by its intracytoplasmic domain (ICD), which is proteolytically cleaved by the intramembrane protease signal peptide peptidase-like (SPPL)2a and subsequently translocates into the nucleus, where it functions as a transcription factor and/or transcriptional coactivator [90–92]. Whether this process occurs in the endolysosomal compartment or on the cell surface and how it is exactly triggered by extracellular MIF has remained partly unclear. A second signaling CD74 pathway involves the association of CD74 with a co-receptor. Depending on the cellular and (patho)physiological context this can be CD44, the initially identified co-receptor of CD74, or one of the MIF chemokine receptors, i.e. CXCR2, CXCR4 or ACKR3/CXCR7 [4, 8, 11, 12]. In our study, we provide evidence for a role of CXCR4, as we obtained evidence from PLA and chemotaxis experiments for CD74/CXCR4 heterocomplex formation to facilitate MIF-elicited chemotaxis of activated T cells. We also obtained evidence for MIF-induced internalization of CD74/CXCR4 heterocomplexes from the surface of T cells.

As mentioned above, CD44 represents another potential co-receptor of CD74 in T cells that is abundantly expressed and is an established activation marker of T cells. Additional studies are necessary to evaluate the functional relevance of CD74/CD44 interactions in T cells [11, 93].

An impaired adaptive immune response linked to sustained T-cell activation and a dysregulated IFN-response is believed to be a significant determinant of COVID-19 progression [30, 32, 94, 95]. Furthermore, accumulating evidence points towards a critical role of MIF as a prognostic marker to predict disease severity and patient outcome in COVID-19 disease. Notably, a recent study by Westmeier et al. investigated MIF receptor expression in CD4^+^ and CD8^+^ T cells in COVID-19 patients with mild and severe disease and observed an increased expression of CD74 in CD4^+^ and CD8^+^ T cells compared to healthy controls [71]. Interestingly, the authors also observed an inducible expression of CXCR2 and CXCR4 upon SARS-CoV-2 infection pointing towards increased susceptibility to MIF-mediated signaling in the course of COVID-19 disease. A characterization of T-cell subpopulations in their study revealed a predominant central and effector memory phenotype of the CD74-expressing T cells that further produced higher cytotoxic molecules and expressed enhanced proliferation markers. In accordance, we observed a significant upregulation of CD74 surface expression on CD4^+^ and CD8^+^ T cells in the severe disease group, when comparing patient cohorts with mild and severe COVID-19 disease. In contrast, no significant differences between both groups regarding CXCR4 expression was observed. CD74 markedly exceeded HLA-DR expression, which showed no significant changes between both cohorts, again confirming an MHC II–independent regulation of CD74 in T cells. Of note, CXCR4 and CD74 expression was also monitored in monocyte subpopulations in the same patient cohort revealing enhanced expression of CD74 in classical monocytes again without significant changes in CXCR4 expression. We speculate that the observed upregulation of CD74 reflects increased COVID-19-induced T-cell activation states, which might enhance susceptibility towards MIF [30, 32]. However, suitability of T-cell CD74 as a potential biomarker for disease progression in COVID-19 and its relevance in other inflammatory or malignant diseases accompanied by broad T-cell activation still needs to be evaluated in future prospective trials. Furthermore, due to the small patient cohort and heterogeneity a subgroup-specific analysis based on factors such as age, gender or comorbidities was not feasible in the presented study.

**Fig. 7:**
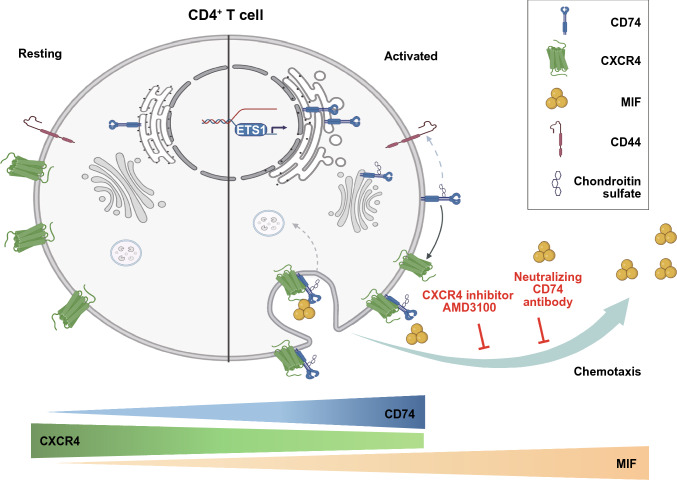
Scheme of the regulation of the MIF receptors CD74 and CXCR4 in resting and activated CD4^+^ T-cell state. During resting state, CD4^+^ T cells express CXCR4 abundantly on the cell surface, while CD74 is constitutively expressed and synthesized intracellularly. Most likely due to its retention signal CD74 resides in the ER with functional circulation in the endolysosomal compartment. Triggered by T-cell activation, CD74 gene expression and protein synthesis is rapidly upregulated in contrast to the initially repressed CXCR4 expression. We speculate, that ETS1 might be involved in the rapid regulation of CD74 in this process. Furthermore, CD74 molecules are post-translationally modified by addition of chondroitin sulfate moieties. This modification enables rapid transport of CD74 towards the cell surface, where it can act as a functional surface receptor for MIF, a proinflammatory cytokine that is secreted during T-cell activation and exerts additional auto- and paracrine effects. In activated CD4^+^ T cells, MIF leads to internalization of CD74/CXCR4 receptor complexes. Both receptors are crucial for MIF-induced chemotaxis, as blockade of either CXCR4 or CD74 abrogates CD4^+^ T-cell migration towards MIF. Scheme was created with BioRender.com (license of the Institute for Stroke and Dementia Research)

In summary, our data identify CD74 as a functional MIF receptor and MHC II-independent activation marker of activated CD4^+^ T cells mediating MIF-driven CD4^+^ T-cell chemotaxis, most likely through complex formation with CXCR4. CD74 and CXCR4 expression levels behave inversely in the course of T-cell activation. Induction of CD74 occurs rapidly upon activation stimulus in naive and memory T cells leading to an activation-induced chondroitin sulfated isoform. We have thus unraveled a previously unrecognized MIF/CD74/CXCR4 signaling pathway in activated human T cells with functional relevance for T-cell motility and potentially other activities of activated T cells (Fig. 7). We confirm high CD74 surface expression in T cells under disease conditions in critically ill COVID-19 patients potentially linking dysregulated CD74 to disease severity. Thus, targeting the dysregulated MIF-CD74 axis might resemble a tractable treatment strategy to interfere with the critical role of MIF in the COVID-19 disease context. To this end, future studies will be needed to clarify whether CD74 could have implications in immunosenescence of T cells with potential relevance for the enhanced susceptibility of the aging population to infections like COVID-19 or reduced responses to vaccinations [96–98].

### Supplementary Information

Below is the link to the electronic supplementary material.

Supplementary file1 Supp. Figure 1. Flow cytometry gating strategies. (A) Gating strategy and cell purity after CD4+ T cell isolation. Visualization of a representative flow cytometry gating consisting of exclusion of debris, dead cells and doublets and verification of CD3+ CD4+ T cell purity after CD4+ T cell isolation from PBMCs of healthy donors. (B) Gating strategy to characterize T cell subpopulations from COVID-19 patients after CD3+ T cell isolation. Visualization of a representative flow cytometry gating consisting of exclusion of debris, dead cells and doublets and validation of CXCR4 and CD74 receptor expression after CD3+ T cell isolation from PBMCs. (C) Gating strategy to characterize monocyte subpopulations from COVID-19 patients. Visualization of a representative flow cytometry gating of monocyte subpopulations as according to Marimuthu et al. with determination of CD74 and CXCR4 expression on classical and non-classical monocytes in PBMC fraction of CD3+ negative cells after CD3+ positive selection. Steps include exclusion of debris, dead cells and doublets, and selecting monocyte subsets by CD16 vs. CD14 plot after exclusion of HLA-DR- natural killer (NK) cells and HLA-DRhighCD14low B cells [34]. Supp. Figure 2. Characterization of CD4+ T cells. (A-C) Validation of in vitro T-cell activation. Surface expression of the naive cell marker CD45RA and CD45RO, as a marker of activated or effector/memory T cells, was measured (A) directly after isolation or (B) after 72 h of in vitro activation using anti-CD3+/anti-CD28+ coated beads. (C) Quantification of RA+RO- (light gray), RA+RO+ (dark gray) and RA-RO+ (black) CD4+ T cells of nine independent experiments (n = 9) is provided as fraction of a whole in the bottom row. (D-G) Alternative quantification of MIF receptor profiling on primary human CD4+ T cells upon activation as shown in Fig. 2. Flow cytometry-based cell surface receptor profiling of the four MIF receptors CD74, CXCR4, CXCR2, and ACKR3, as indicated, on purified human CD4+ T cells before (0 h) and after 72 h of in vitro T-cell activation. Comparison and quantification of the cell surface median fluorescence intensity (MFI) for each of the four receptors (E, n=22; F, n=11; G, n=9; H, n=6). Statistical differences were analyzed by Wilcoxon matched-pairs signed-rank test and indicated by actual P values. Supp. Figure 3. Renewal rates and protein dynamics of selected proteins. (A-B) Re-analysis of publicly available proteomic data of memory CD4+ T cells after 5 d of different activation and cytokine polarization conditions (resting: no activation, no added cytokines; Th0: control with no added cytokines; Th1: IL-12, anti-human IL-4 antibody; TH2: IL-4, anti-human IFN-γ antibody, Th17: IL-6, IL-23, IL-1β, TGF-β1, anti-human IL-4 antibody, anti-human IFN-γ antibody; iTreg: TGF-β1, IL-2; IFN-β-stimulated group) according to Cano-Gamez et al. regarding protein abundance of (A) CD74 and (B) CXCR4 [28]. Statistical differences were analyzed by one-way ANOVA with test for multiple comparisons. (C-D) Comparison of protein renewal rates in resting naive (blue) vs. resting memory (orange) CD4+ T cells. Fraction of newly synthesized protein calculated from LC-MS/MS analysis of pulsed SILAC of CD4+ T cells. Cells were analyzed after 0 h, 6 h, 12 h, 24 h and 48 h in culture. (C) Exemplary representation of fast (ETS1), intermediate (CD3E) and slow (GAPDH) renewal rate. (D) Renewal rates of CXCR4 (left), CD44 (middle) and MIF (right). (E-G) Time course of protein expression per cell upon activation of naive CD4+ T cells. Label-free quantification of proteins via the MaxQuant algorithm without and after 6 h, 12 h, 24 h, 48 h, 72 h, 96 h, 120 h and 144 h of in vitro activation. Proteins identified by MS/MS (black) or matching (orange). Estimation of copy number per cell based on protein mass of cell. (E-G) Comparative presentation of established (E) fast (CD69), (F) intermediate (IL2Rα/CD25) and (G) late (HLA-DRA) T-cell activation markers. Data in (C-G) retrieved and re-analyzed from Wolf et al. [36]. Supp. Figure 4. CIITA interaction network. Visualization of the ten proteins most strongly associated with functional CIITA interaction as predicted by the STRING database [42]. Supp. Figure 5. Dose curves and controls of the 3D chemotaxis experiments. (A-B) MIF dose-dependently induces chemotaxis of activated CD4+ T cells. Trajectory plots (x, y = 0 at time 0 h) and corresponding quantification of migrated activated CD4+ T cells in a three-dimensional (3D) aqueous collagen-gel matrix towards MIF chemoattractant gradients (MIF concentrations: 100 ng/ml – 800 ng/ml as indicated, -: control medium). Plotted is the calculated forward migration index (FMI, mean ± SD) based on manual tracking of at least 30 individual cells per treatment (n=1). Statistical differences were analyzed by Kruskal-Wallis test with Dunn post-hoc test. (C-D) Inhibitor-only controls of the presented chemotaxis experiment in Fig. 5. Representative trajectory plots and quantification of migrated activated CD4+ T cells in the presence of a CD74 neutralizing antibody, a corresponding isotype control (IgG) or the CXCR4 receptor inhibitor AMD3100. Cell motility in (A-D) was monitored by time-lapse microscopy for 2 h at 37°C, images were obtained every minute using the Leica DMi8 microscope. Single cell tracking was performed of 30 cells per experimental group. The blue crosshair indicates the cell population’s center of mass after migration. Quantification of the 3D chemotaxis experiment in (C-D) showing no chemotactic effects of the inhibitors alone. Plotted is the calculated forward migration index (FMI, mean ± SD) based on manual tracking of at least 30 individual cells per treatment (n=3-4). Statistical differences were analyzed by Kruskal-Wallis test with Dunn post-hoc test. Supp. Figure 6. Characterization of monocyte subpopulations from COVID-19 patients. (A-C) Comparison of monocyte subpopulations in patients with mild and severe COVID-19 disease. Percentages of monocyte subpopulations in patients with mild (WHO 1-3, 18 patients) vs. severe (WHO ≥ 5, 12 patients) COVID-19 disease determined via flow cytometry as described in Supp. Fig. 1C. (D-E) Upregulation of CD74 surface expression in classical monocytes of critically ill COVID-19 patients. CD74 and CXCR4 surface expression in classical monocyte subpopulation in mild vs. severe COVID-19 disease patients. Bar charts in (A-E) show means ± SD with individual datapoints representing independent patients. Statistical differences were analyzed by unpaired t test for A, C, D and Mann-Whitney U test for B and F and indicated by actual P values. Supp. Table 1. List of antibodies used for flow cytometry experiments with additional information (PDF 1787 KB)

Supplementary file2 Supp. Table 2. List of potential transcription factor binding sites upstream from the CD74 gene locus. Potential transcription factor binding sites at a maximum distance of 500 bp from the CD74 gene locus were identified in the Gene Transcription Regulation Database (GTRD) [40]. See accompanying excel file for detailed list. Supp. Table 3. List of predicted transcription factors involved in CD74 gene expression. Potential transcription factors involved in the transcriptional regulation of CD74 identified using the PathwayNet database [41]. Shown are genes with a relationship confidence of more than 0.1. Yellow marked are CIITA-associated transcription factors that were identified in Supp. Fig. 4. Orange marked are genes with no binding site within 500 bp of the CD74 gene as identified in Supp. Table 2. See accompanying excel file (XLSX 82 KB)

## Data Availability

All data and materials as well as software application information are available in the manuscript, the supplementary information, or are available from the corresponding authors upon reasonable request. The dataset published by Szabo et al., which was re-analyzed during the current study is publicly available on the gene expression omnibus (GEO) under accession number GSE126030 [35]. Plots were generated using the Single Cell Expression Atlas of the European Bioinformatics Institute (EBI) of the European Molecular Biology Laboratory (EMBL) (https://www.ebi.ac.uk/gxa/sc/experiments/E-HCAD-8/results/tsne, last visited 20th of December, 2023). Secondly, a bulk-RNAseq data set together with the according proteomic data as recently published by Cano-Gamez et al. was re-analyzed [28]. The RNAseq raw data were accessed via the Open Targets website (https://www.opentargets.org/projects/effectorness) and subsequently re-analyzed as described in the manuscript. The full analysis code is published on GitHub (https://github.com/SimonE1220/CD74Tcelldiff). The available proteomic raw data were accessed via the Proteomics Identifications Database (PRIDE) under the accession number PXD015315. Additionally, a data set published by Wolf et al. was re-analyzed [39]. The data-set is publicly accessible in the GEO with accession number GSE147229 and GSE146787 or via www.immunomics.ch (last visited 7th of December, 2023).
