# Supplementary material for: CD74 is a functional MIF receptor on activated CD4+ T cells
Source: Cell Mol Life Sci. 2024 Jul 11;81(1):296. doi: 10.1007/s00018-024-05338-5 (PMC11335222; doi:10.1007/s00018-024-05338-5)
Supplement: Supplementary file 1 — Supplementary file1 Supp. Figure 1. Flow cytometry gating strategies. (A) Gating strategy and cell purity after CD4+ T cell isolation. Visualization of a representative flow cytometry gating consisting of exclusion of debris, dead cells and doublets and verification of CD3+ CD4+ T cell purity after CD4+ T cell isolation from PBMCs of healthy donors. (B) Gating strategy to characterize T cell subpopulations from COVID-19 patients after CD3+ T cell isolation. Visualization of a representative flow cytometry gating consisting of exclusion of debris, dead cells and doublets and validation of CXCR4 and CD74 receptor expression after CD3+ T cell isolation from PBMCs. (C) Gating strategy to characterize monocyte subpopulations from COVID-19 patients. Visualization of a representative flow cytometry gating of monocyte subpopulations as according to Marimuthu et al. with determination of CD74 and CXCR4 expression on classical and non-classical monocytes in PBMC fraction of CD3+ negative cells after CD3+ positive selection. Steps include exclusion of debris, dead cells and doublets, and selecting monocyte subsets by CD16 vs. CD14 plot after exclusion of HLA-DR- natural killer (NK) cells and HLA-DRhighCD14low B cells [34]. Supp. Figure 2. Characterization of CD4+ T cells. (A-C) Validation of in vitro T-cell activation. Surface expression of the naive cell marker CD45RA and CD45RO, as a marker of activated or effector/memory T cells, was measured (A) directly after isolation or (B) after 72 h of in vitro activation using anti-CD3+/anti-CD28+ coated beads. (C) Quantification of RA+RO- (light gray), RA+RO+ (dark gray) and RA-RO+ (black) CD4+ T cells of nine independent experiments (n = 9) is provided as fraction of a whole in the bottom row. (D-G) Alternative quantification of MIF receptor profiling on primary human CD4+ T cells upon activation as shown in Fig. 2. Flow cytometry-based cell surface receptor profiling of the four MIF receptors CD74, CXCR4, CXCR2, and ACKR3, as indi [file 18_2024_5338_MOESM1_ESM.pdf]

Supplementary File

CD74 is a functional MIF receptor on activated CD4$^+$ T cells

Lin Zhang[1, *], Iris Woltering[1, *], Mathias Holzner[1], Markus Brandhofer[1], Carl-Christian Schaefer[1], Genta Bushati[1], Simon Ebert[1], Bishan Yang[1], Maximilian Muenchhoff[2,3,4], Johannes C. Hellmuth[4,5], Clemens Scherer[4,6], Christian Wichmann[7], David Effinger[8,9], Max Hübner[8,9], Omar El Bounkari[1], Patrick Scheiermann[8], Jürgen Bernhagen[1,10, #], Adrian Hoffmann[1,8,10, #]

[1]Division of Vascular Biology, Institute for Stroke and Dementia Research (ISD), LMU University Hospital (LMU Klinikum), Ludwig-Maximilians-Universität (LMU) München, Feodor-Lynen-Straße 17, 81377 Munich, Germany; [2]Max-von-Pettenkofer Institute and Gene Center, Virology, National Reference Center for Retroviruses, Ludwig-Maximilians-Universität (LMU) München, Munich, Germany; [3]German Center for Infection Research (DZIF), Partner Site Munich, Munich, Germany; [4]COVID-19 Registry of the LMU Munich (CORKUM), LMU University Hospital, Ludwig-Maximilians-Universität (LMU) München, Munich, Germany; [5]Department of Medicine III, LMU University Hospital, Ludwig-Maximilians-Universität (LMU) München, Munich, Germany; [6] Department of Medicine I, LMU University Hospital, Ludwig-Maximilians-Universität (LMU) München, Munich, Germany; [7]Division of Transfusion Medicine Cell Therapeutics and Haemostaseology, LMU University Hospital, Ludwig-Maximilians-Universität (LMU) München, Munich, Germany; [8]Department of Anaesthesiology, LMU University Hospital, Ludwig-Maximilians-Universität (LMU) München, Marchioninistraße 15, 81377 Munich, Germany; [9]Walter Brendel Centre of Experimental Medicine, Ludwig-Maximilians-Universität (LMU) München, Munich, Germany; [10]German Centre of Cardiovascular Research (DZHK), Partner Site Munich Heart Alliance, Munich, Germany.

*Lin Zhang and Iris Woltering are equally contributing first authors.

[#]Correspondence:

Professor Jürgen Bernhagen, PhD
Chair of Vascular Biology, Institute for Stroke and Dementia Research (ISD)
LMU University Hospital (LMU Klinikum), Ludwig-Maximilians-Universität (LMU) München
Feodor-Lynen-Straße 17, 81377 Munich, Germany
Tel.: 0049-89-4400-46151
E-Mail: juergen.bernhagen@med.uni-muenchen.de

Adrian Hoffmann, MD
Department of Anaesthesiology, LMU University Hospital
Ludwig-Maximilians-Universität (LMU) München
Marchioninistraße 15, 81377 Munich, Germany
Tel.: 0049-894400-16-81177
E-Mail: adrian.hoffmann@med.uni-muenchen.de

Table of Contents

Supplementary Figures

Supplementary Figure 1

Supplementary Figure 2

Supplementary Figure 3

Supplementary Figure 4

Supplementary Figure 5

Supplementary Figure 6

Supplementary Tables

Supplementary Table 1

Supplementary Table 2

Supplementary Table 3

References

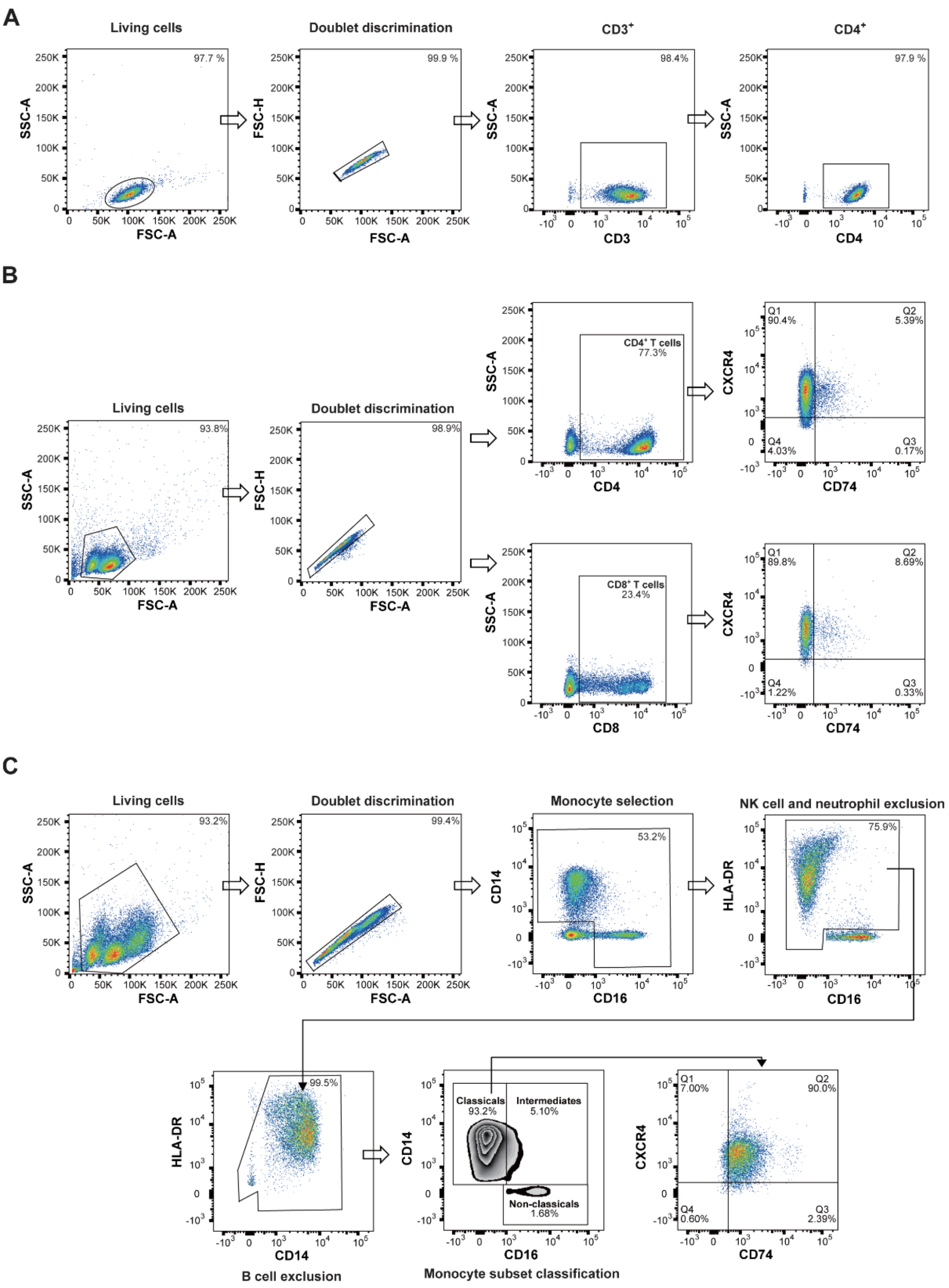

Supplementary Figure 1. Flow cytometry gating strategies. **A** Gating strategy and cell purity after CD4⁺ T-cell isolation. Visualization of a representative flow cytometry gating consisting of exclusion of debris, dead cells and doublets and verification of CD3⁺ CD4⁺ T-cell purity after CD4⁺ T-cell isolation from PBMCs of healthy donors. **B** Gating strategy to characterize T-cell subpopulations from COVID-19 patients after CD3⁺ T-cell isolation. Visualization of a representative flow cytometry gating consisting of exclusion of debris, dead cells and doublets and validation of CXCR4 and CD74 receptor expression after CD3⁺ T-cell isolation from PBMCs. **C** Gating strategy to characterize monocyte subpopulations from COVID-19 patients.

Visualization of a representative flow cytometry gating of monocyte subpopulations according to Marimuthu et al with determination of CD74 and CXCR4 expression on classical and non-classical monocytes in PBMC fraction of $CD3^+$-negative cells after $CD3^+$-positive selection. Steps include exclusion of debris, dead cells and doublets, and selecting monocyte subsets by CD16 vs. CD14 plot after exclusion of $HLA\text{-}DR^-$ natural killer (NK) cells and $HLA\text{-}DR^{high}CD14^{low}$ B cells [1].

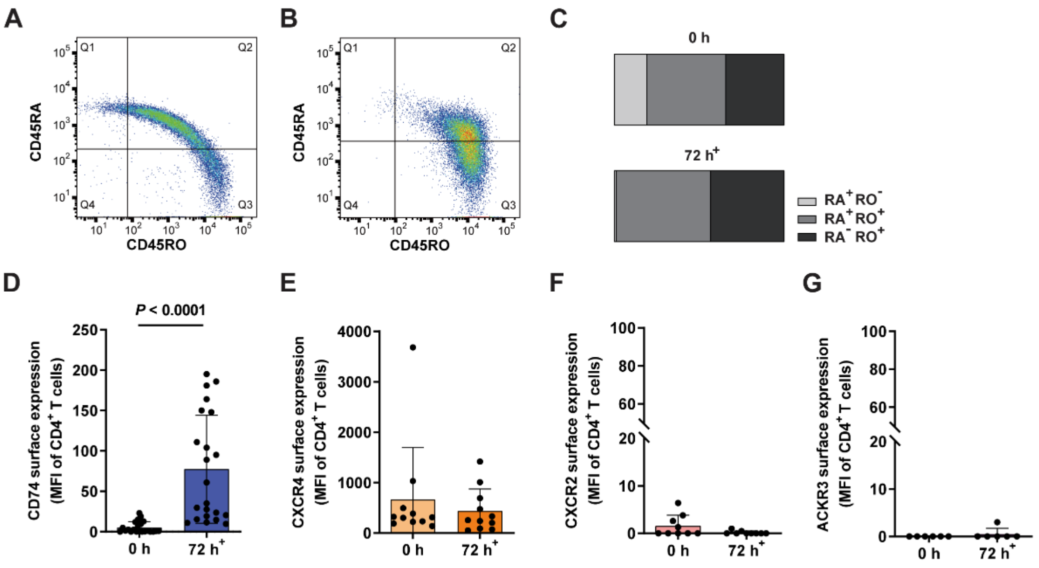

Supplementary Figure 2. Characterization of CD4$^+$ T cells. **A**-**C** Validation of *in vitro* T-cell activation. Surface expression of the naive cell marker CD45RA and CD45RO, as a marker of activated or effector/memory T cells, was measured **A** directly after isolation or **B** after 72 h of *in vitro* activation using anti-CD3$^+$/anti-CD28$^+$ coated beads. **C** Quantification of RA$^+$RO$^-$ (light gray), RA$^+$RO$^+$ (dark gray) and RA$^-$RO$^+$ (black) CD4$^+$ T cells of nine independent experiments (n = 9) is provided as fraction of a whole in the bottom row. **D**-**G** Alternative quantification of MIF receptor profiling on primary human CD4$^+$ T cells upon activation as shown in Fig. 2. Flow cytometry-based cell surface receptor profiling of the four MIF receptors CD74, CXCR4, CXCR2, and ACKR3, as indicated, on purified human CD4$^+$ T cells before (0 h) and after 72 h of *in vitro* T-cell activation. Comparison and quantification of the cell surface median fluorescence intensity (MFI) for each of the four receptors (**E**, n=22; **F**, n=11; **G**, n=9; **H**, n=6). Statistical differences were analyzed by Wilcoxon matched-pairs signed-rank test and indicated by actual *P* values.

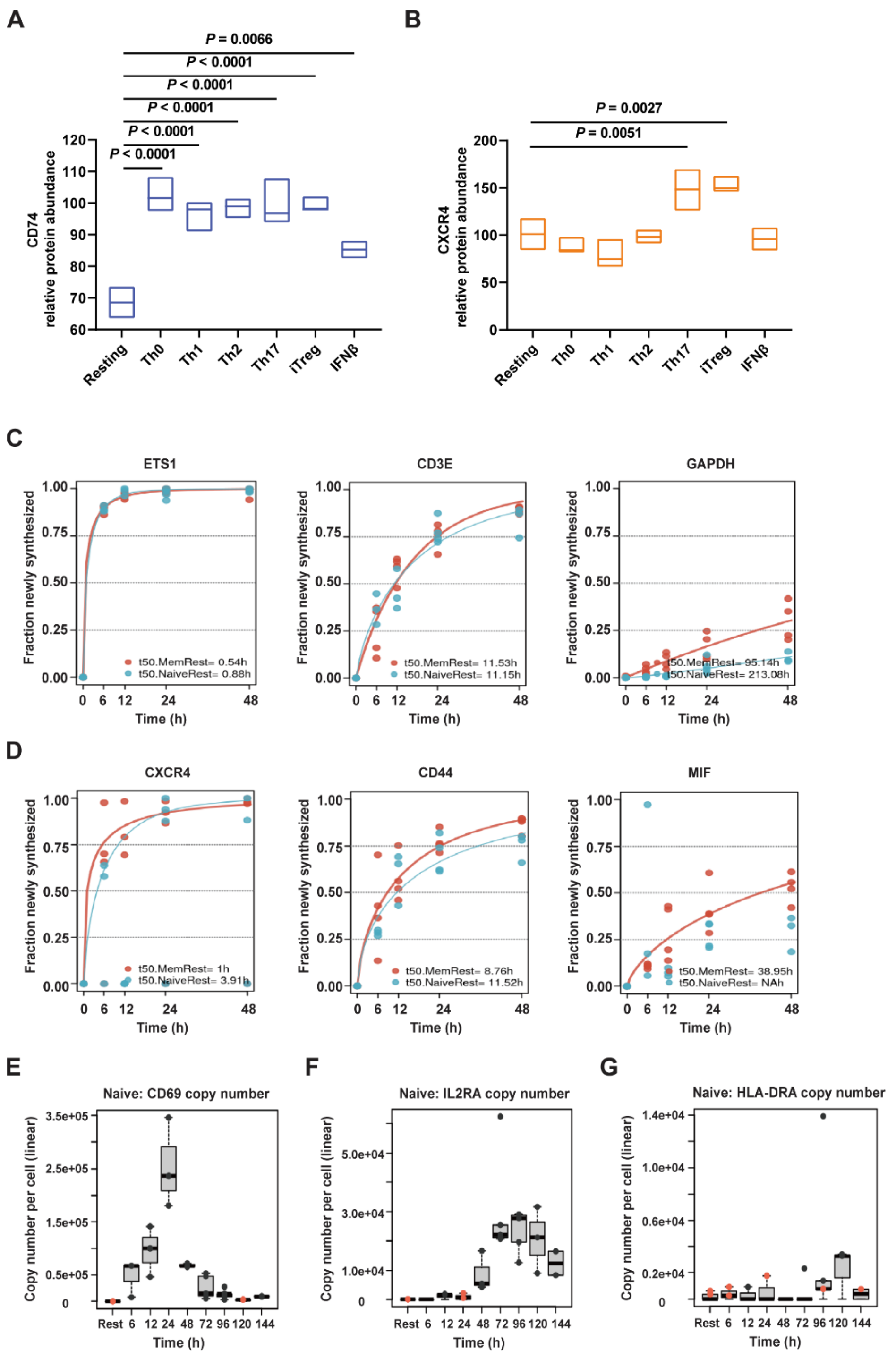

Supplementary Figure 3. Renewal rates and protein dynamics of selected proteins. **A**-**B** Re-analysis of publicly available proteomic data of memory CD4+ T cells after 5 d of different activation and cytokine polarization conditions (resting: no activation, no added cytokines; Th0: control with no added cytokines; Th1: IL-12, anti-human IL-4 antibody; TH2: IL-4, anti-human IFN-γ antibody, Th17: IL-6, IL-23, IL-1β, TGF-β1, anti-human IL-4 antibody, anti-human IFN-γ antibody; iTreg: TGF-β1, IL-2; IFN-β-stimulated group) according to Cano-Gamez et al. regarding protein abundance of **A** CD74 and **B** CXCR4 [2]. Statistical differences were analyzed by one-way ANOVA with post-hoc multiple comparisons test. **C**-**D** Comparison of protein

renewal rates in resting naive (blue) vs. resting memory (orange) $CD4^+$ T cells. Fraction of newly synthesized protein calculated from LC-MS/MS analysis of pulsed SILAC of $CD4^+$ T cells. Cells were analyzed after 0 h, 6 h, 12 h, 24 h and 48 h in culture. **C** Exemplary representation of fast (ETS1), intermediate (CD3E) and slow (GAPDH) renewal rate. **D** Renewal rates of CXCR4 (left), CD44 (middle) and MIF (right). **E**-**G** Time course of protein expression per cell upon activation of naive $CD4^+$ T cells. Label-free quantification of proteins via the MaxQuant algorithm without and after 6 h, 12 h, 24 h, 48 h, 72 h, 96 h, 120 h and 144 h of *in vitro* activation. Proteins identified by MS/MS (black) or matching (orange). Estimation of copy number per cell based on protein mass of cell. **E-G** Comparative presentation of established **E** fast (CD69), **F** intermediate (IL2Rα/CD25) and **G** late (HLA-DRA) T-cell activation markers. Data in **C**-**G** retrieved and re-analyzed from Wolf et al [3].

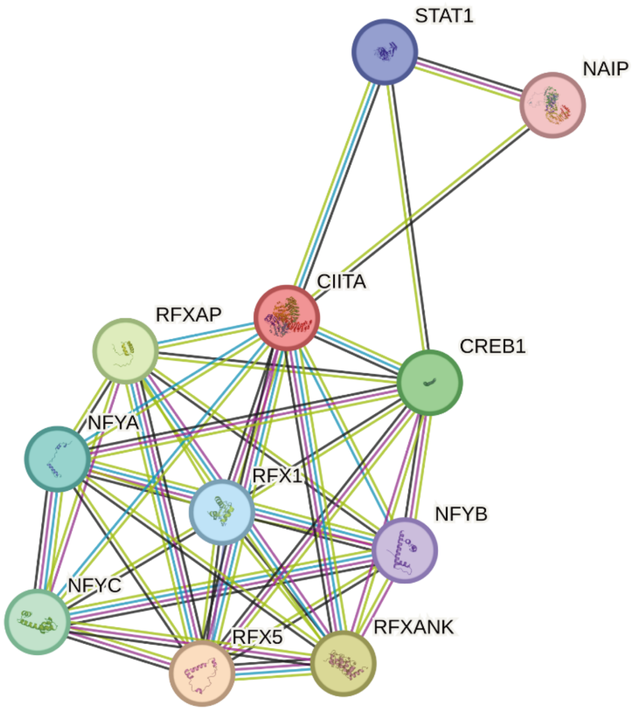

Supplementary Figure 4. CIITA interaction network. Visualization of the ten proteins most strongly associated with functional CIITA interaction as predicted by the STRING database [4].

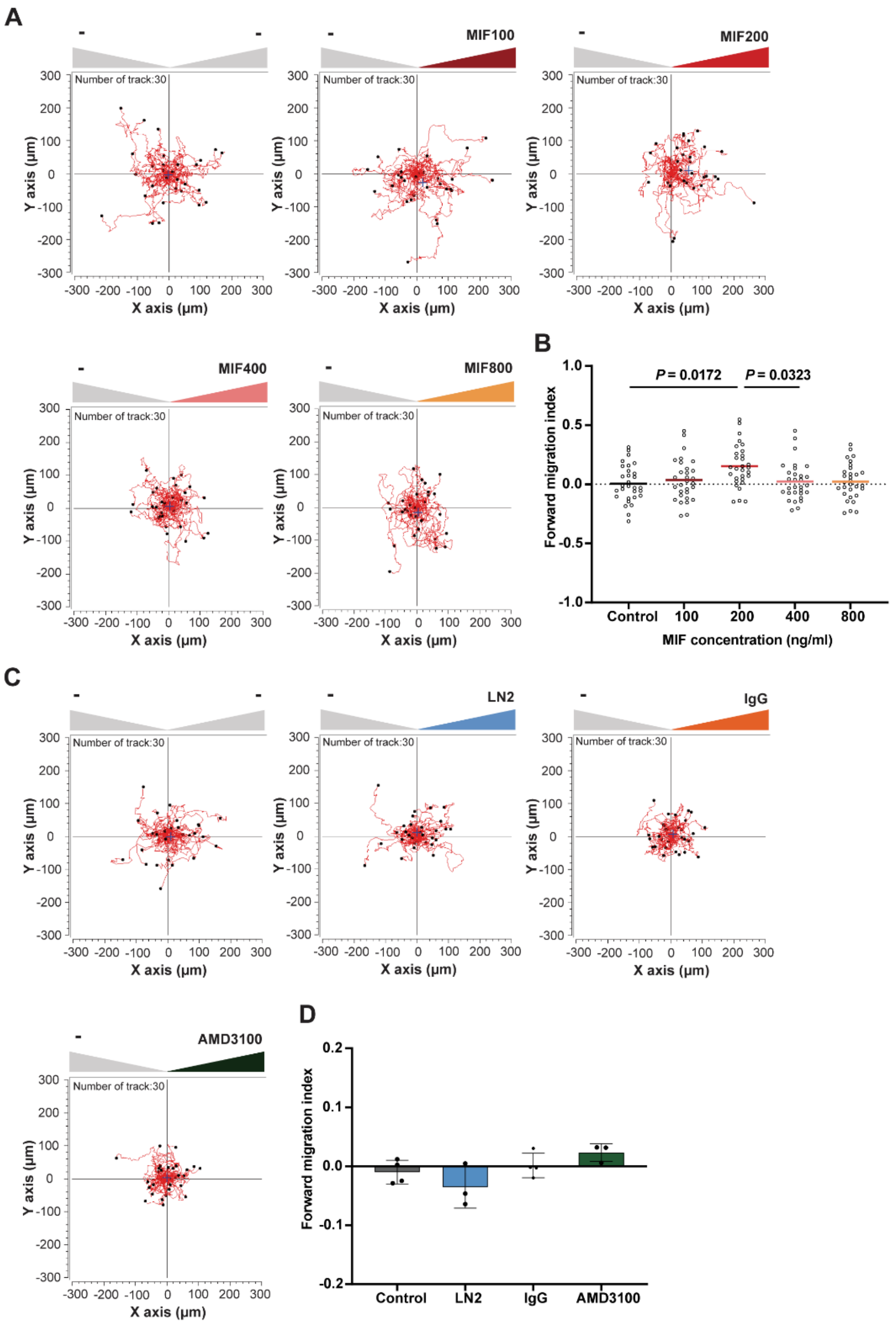

Supplementary Figure 5. Dose curves and controls of the 3D chemotaxis experiments. **A-B** MIF dose-dependently induces chemotaxis of activated CD4$^+$ T cells. Trajectory plots (x, y = 0 at time 0 h) and corresponding quantification of migrated activated CD4$^+$ T cells in a three-dimensional (3D) aqueous collagen-gel matrix towards MIF chemoattractant gradients (MIF concentrations: 100 ng/ml – 800 ng/ml as indicated, -: control medium). Plotted is the calculated forward migration index (FMI, mean ± SD) based on manual tracking of at least 30 individual cells per treatment (n=1). Statistical differences were analyzed by Kruskal-Wallis test with Dunn post-hoc test. **C-D** Inhibitor-only controls of the presented chemotaxis experiment in Fig. 5. Representative trajectory plots and quantification of migrated activated CD4$^+$ T cells in the

presence of a CD74 neutralizing antibody, a corresponding isotype control (IgG) or the CXCR4 receptor inhibitor AMD3100. Cell motility in **A**-**D** was monitored by time-lapse microscopy for 2 h at 37°C, images were obtained every minute using the Leica DMi8 microscope. Single cell tracking was performed of 30 cells per experimental group. The blue crosshair indicates the cell population's center of mass after migration. Quantification of the 3D chemotaxis experiment in **C**-**D** showing no chemotactic effects of the inhibitors alone. Plotted is the calculated forward migration index (FMI, mean ± SD) based on manual tracking of at least 30 individual cells per treatment (n=3-4). Statistical differences were analyzed by Kruskal-Wallis test with Dunn's post-hoc test.

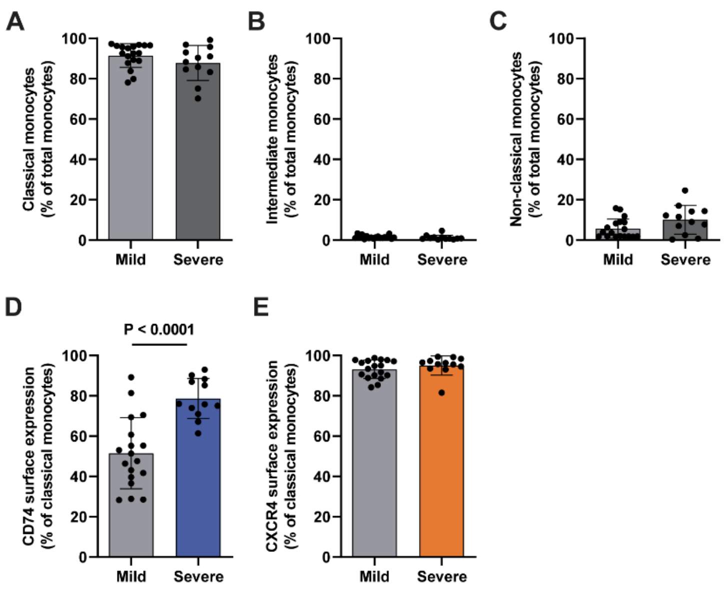

Supplementary Figure 6. Characterization of monocyte subpopulations from COVID-19 patients. **A**-**C** Comparison of monocyte subpopulations in patients with mild and severe COVID-19 disease. Percentages of monocyte subpopulations in patients with mild (WHO 1-3, 18 patients) vs. severe (WHO ≥ 5, 12 patients) COVID-19 disease determined via flow cytometry as described in Supp. Fig. 1C. **D**-**E** Upregulation of CD74 surface expression in classical monocytes of critically ill COVID-19 patients. CD74 and CXCR4 surface expression in classical monocyte subpopulation in mild vs. severe COVID-19 disease patients. Bar charts in **A**-**E** show means ± SD with individual datapoints representing independent patients. Statistical differences were analyzed by unpaired t test for **A**, **C**, **D** and Mann-Whitney U test for **B** and **F** and indicated by actual *P* values.

Supplementary Table 1. List of antibodies used for flow cytometry experiments with additional information.

Antibody	Dilution	Cat Nummber	Company
Anti- hCD14- Pacific Blue	1:100	301828	BioLegend (San Diego, USA)
Anti- hCD16- PerCP	1:100	360720	BioLegend (San Diego, USA)
Anti- hCD3- APC	1:100	300412	BioLegend (San Diego, USA)
Anti- hCD4- PE	1:100	130-113-214	Miltenyi Biotec (Bergisch Gladbach, Germany)
Anti- hCD45 RA- PerCP	1:100	304156	BioLegend (San Diego, USA)
Anti- hCD45 RO- APC	1:100	304210	BioLegend (San Diego, USA)
Anti- hCD74- FITC	1:100	555540	BD Biosciences (Franklin Lakes, USA)
Anti- hCD8- Pacific Blue	1:100	344718	BioLegend (San Diego, USA)
Anti- hCXCR2- FITC	1:100	FAB331F	R&D Systems (Minneapolis, USA)
Anti- hCXCR4- APC	1:100	305510	BioLegend (San Diego, USA)
Anti- hCXCR4- APC/Cy 7	1:100	306528	BioLegend (San Diego, USA)
Anti- hCXCR7- PE	1:100	FAB4227P	R&D Systems (Minneapolis, USA)
Anti- hHLA-DR- PE	1:100	307606	BioLegend (San Diego, USA)
Anti- hHLA-DR- PerCP	1:100	307628	BioLegend (San Diego, USA)

Supplementary Table 2. List of potential transcription factor binding sites upstream from the *CD74* gene locus. Potential transcription factor binding sites at a maximum distance of 500 bp from the *CD74* gene locus were identified in the Gene Transcription Regulation Database (GTRD) [5]. See accompanying excel file for detailed list.

Supplementary Table 3. List of predicted transcription factors involved in CD74 gene expression. Potential transcription factors involved in the transcriptional regulation of CD74 identified using the PathwayNet database [6]. Shown are genes with a relationship confidence of more than 0.1. Yellow marked are CIITA-associated transcription factors that were identified in Supp. Fig. 4. Orange marked are genes with no binding site within 500 bp of the CD74 gene as identified in Supp. Table 2. See accompanying excel file.

References cited in Supplementary file.

1. Marimuthu R, Francis H, Dervish S, Li SCH, Medbury H, and Williams H (2018) Characterization of human monocyte subsets by whole blood flow cytometry analysis. *J Vis Exp.* https://doi.org/10.3791/57941.
2. Cano-Gamez E, Soskic B, Roumeliotis TI, So E, Smyth DJ, Baldrighi M, Willé D, Nakic N, Esparza-Gordillo J, Larminie CGC, Bronson PG, Tough DF, Rowan WC, Choudhary JS, and Trynka G (2020) Single-cell transcriptomics identifies an effectorness gradient shaping the response of CD4(+) T cells to cytokines. *Nat Commun* 11, 1801. https://doi.org/ 10.1038/s41467-020-15543-y.
3. Wolf T, Jin W, Zoppi G, Vogel IA, Akhmedov M, Bleck CKE, Beltraminelli T, Rieckmann JC, Ramirez NJ, Benevento M et al (2020) Dynamics in protein translation sustaining T cell preparedness. *Nat Immunol* 21:927–937. https://doi.org/10.1038/s41590- 020- 0714-5.
4. Szklarczyk D, Gable AL, Nastou KC, Lyon D, Kirsch R, Pyysalo S, Doncheva NT, Legeay M, Fang T, Bork P, Jensen LJ, and von Mering C (2021) The STRING database in 2021: customizable protein-protein networks, and functional characterization of user-uploaded gene/measurement sets. *Nucleic Acids Res* 49, D605-d612. https://doi.org/-10.1093/nar/gkaa1074.
5. Yevshin I, Sharipov R, Kolmykov S, Kondrakhin Y, and Kolpakov F (2019) GTRD: a database on gene transcription regulation-2019 update. *Nucleic Acids Res* 47, D100-d105. https://doi. org/10.1093/nar/gky1128.
6. Wong AK, Park CY, Greene CS, Bongo LA, Guan Y, and Troyanskaya OG (2012) IMP: a multi-species functional genomics portal for integration, visualization and prediction of protein functions and networks. *Nucleic Acids Res* 40, W484-490. https://doi.org/10.1093/ nar/gks458.
